# Supplementary material for: Metabolic reprogramming of cancer cells by JMJD6-mediated pre-mRNA splicing associated with therapeutic response to splicing inhibitor
Source: eLife. 2024 Mar 15;12:RP90993. doi: 10.7554/eLife.90993 (PMC10942784; doi:10.7554/eLife.90993)

Figure 2—supplement 1

<https://hgserver1.amc.nl/cgi-bin/r2/main.cgi>

Dataset: [GSE45547](http://www.ncbi.nlm.nih.gov/geo/query/acc.cgi?acc=GSE45547)


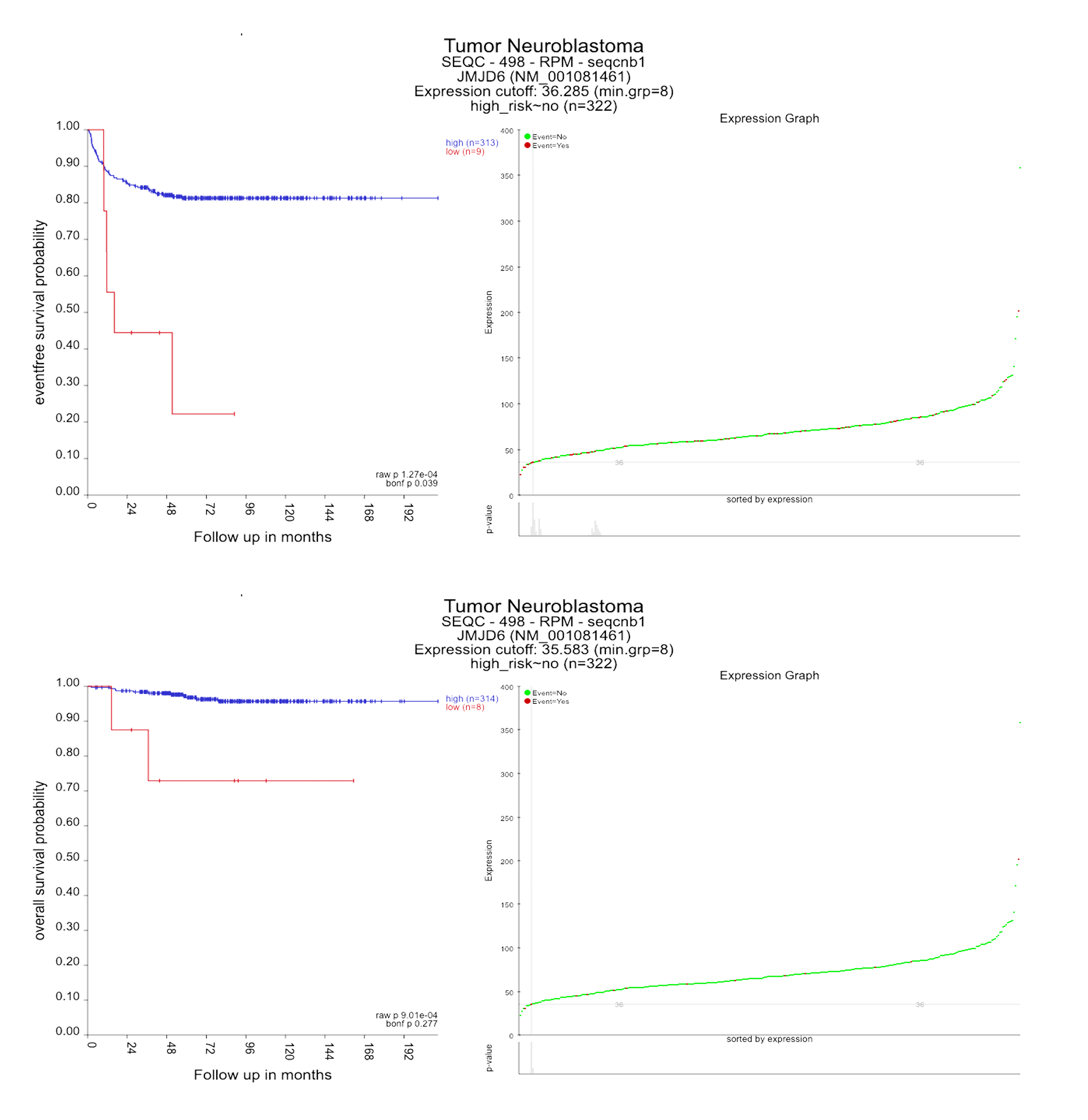


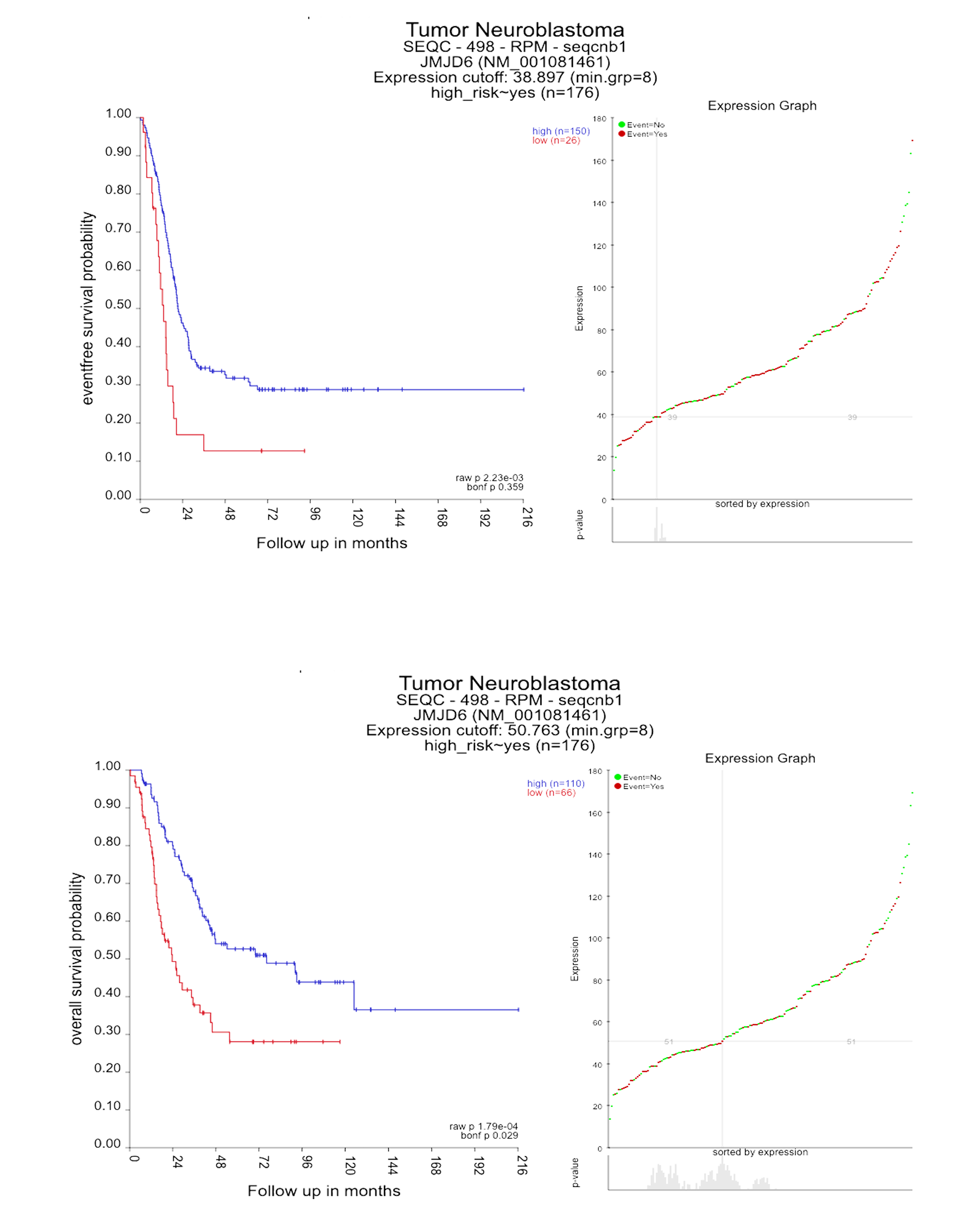

Supplement: Source data 1. [file elife-90993-data1.zip › Figure 2-supplement 1-data source.docx]
