# Supplementary material for: Metabolic reprogramming of cancer cells by JMJD6-mediated pre-mRNA splicing associated with therapeutic response to splicing inhibitor
Source: eLife. 2024 Mar 15;12:RP90993. doi: 10.7554/eLife.90993 (PMC10942784; doi:10.7554/eLife.90993)
Supplement: Source data 1. [file elife-90993-data1.zip › Figure 2 data source.pdf]

Figure 2B
https://hgserver1.amc.nl/cgi-bin/r2/main.cgi
Dataset: GSE62564

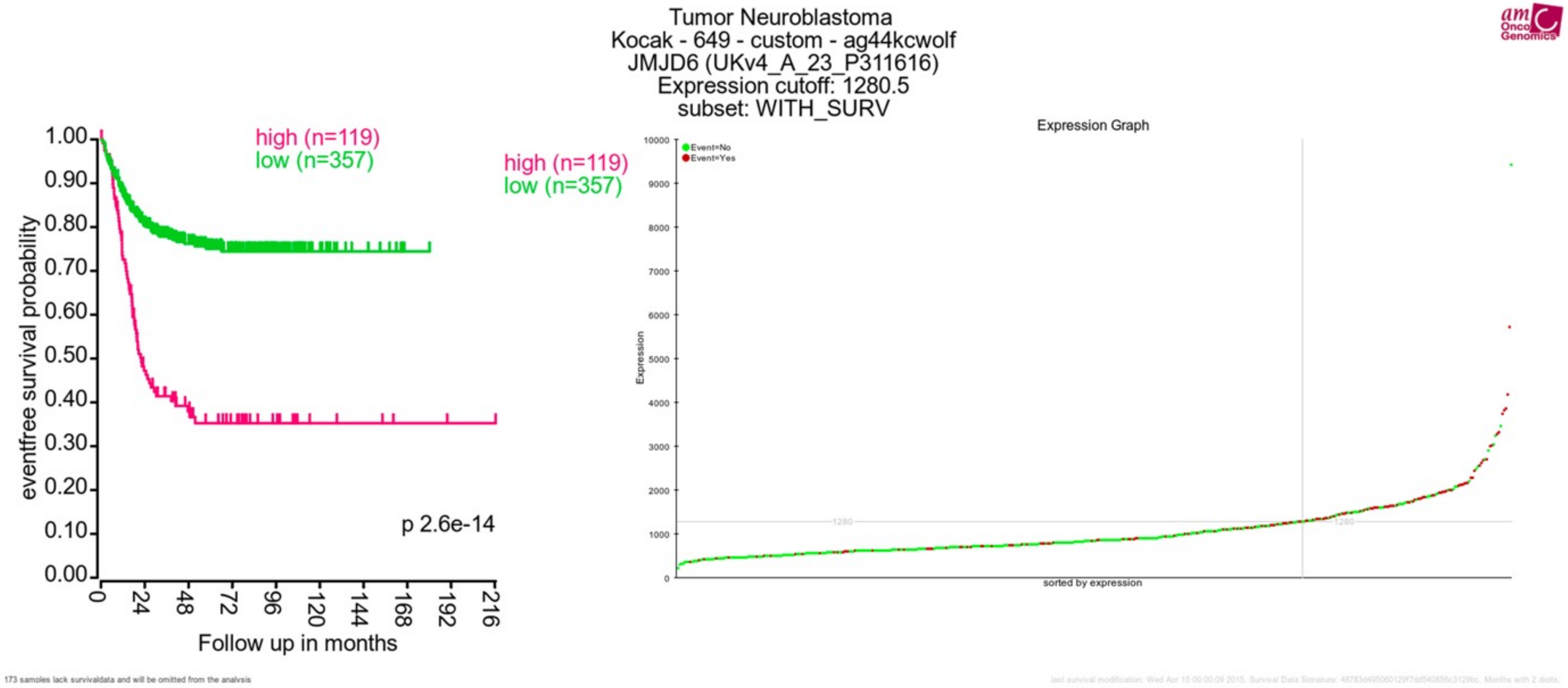

Figure 2C- SKNAS

JMJD6

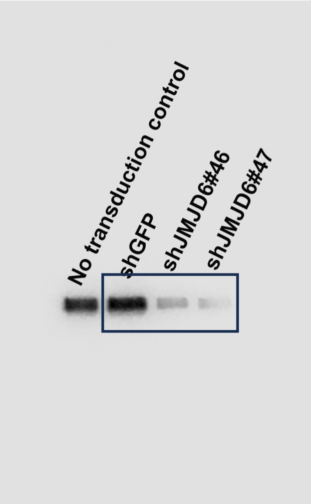

GAPDH

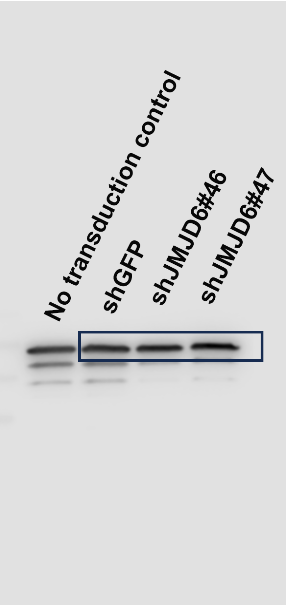

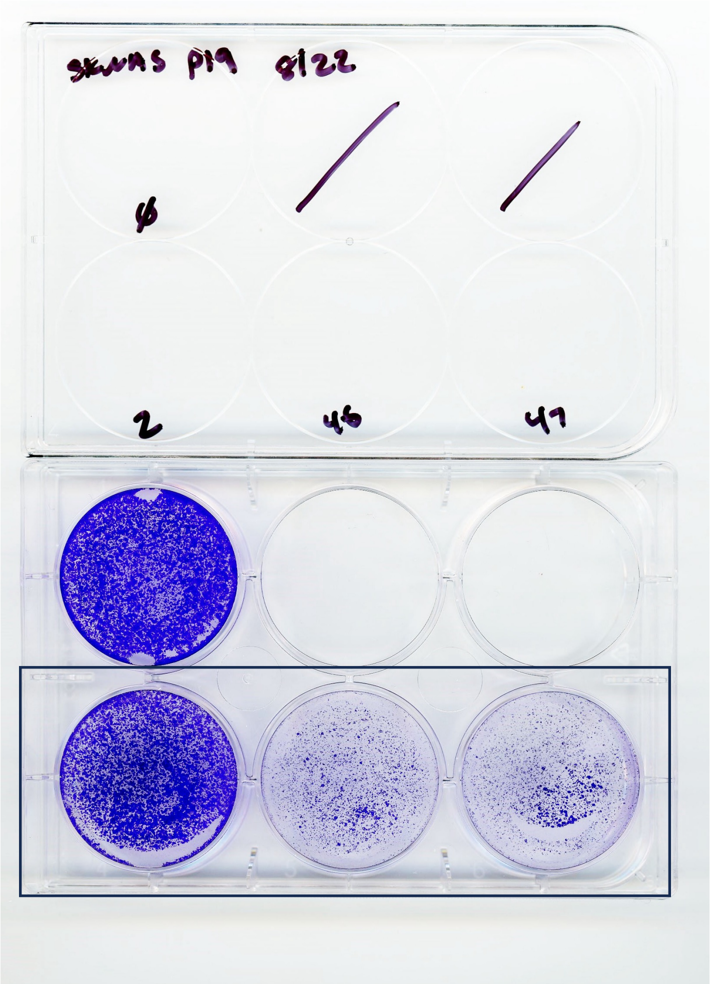

Ø = non transduction control
2 = shGFP
46 = shJMJD6#46
47 = shJMJD6#47

Figure 2C- BE2C

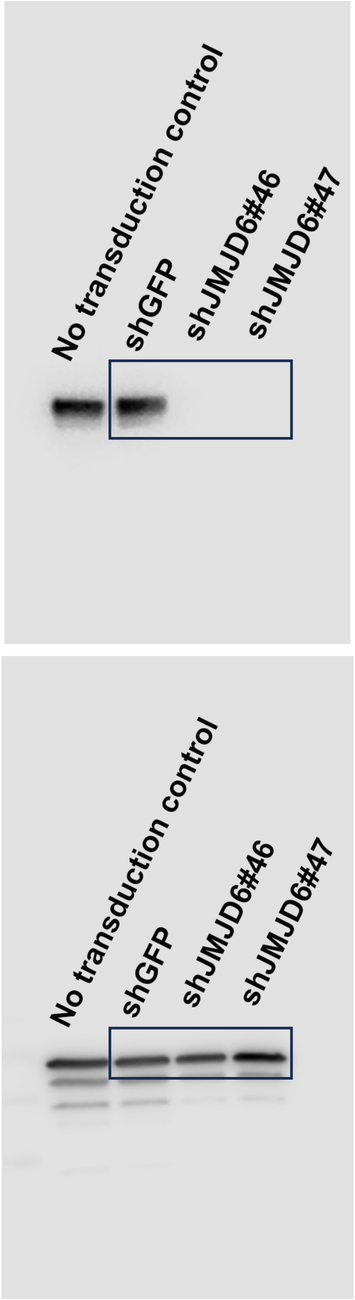

JMJD6

GAPDH

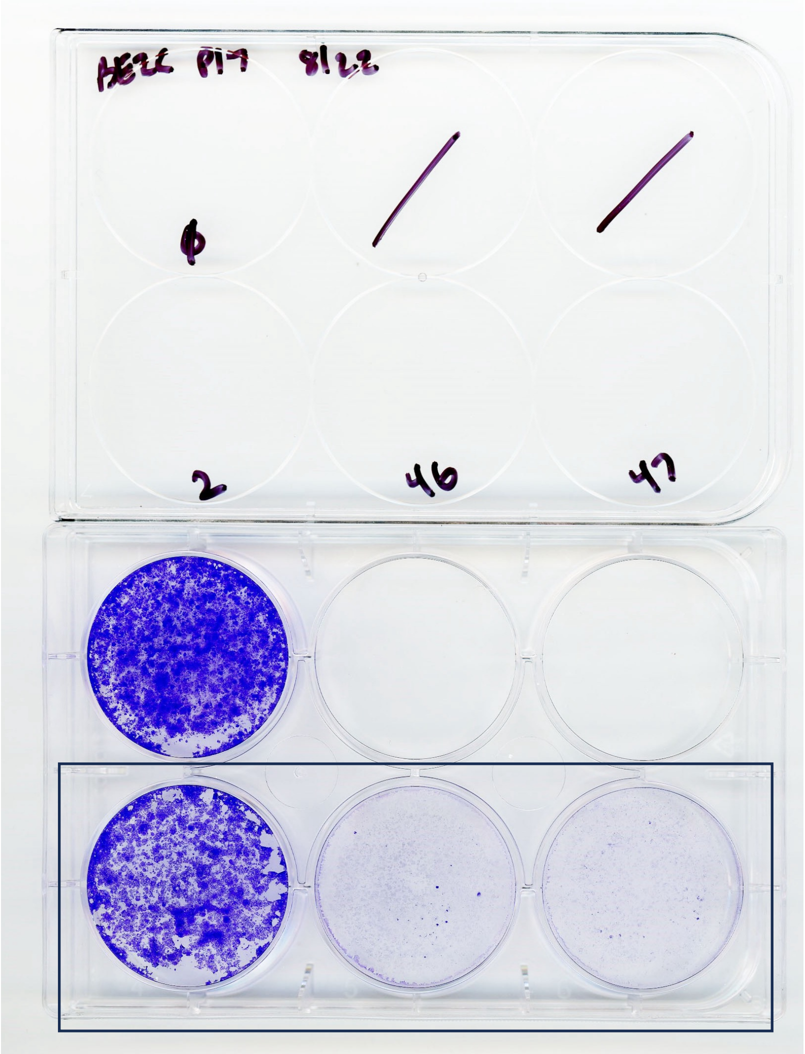

Ø = non transduction control
2 = shGFP
46 = shJMJD6#46
47 = shJMJD6#47

Figure 2C- KELLY

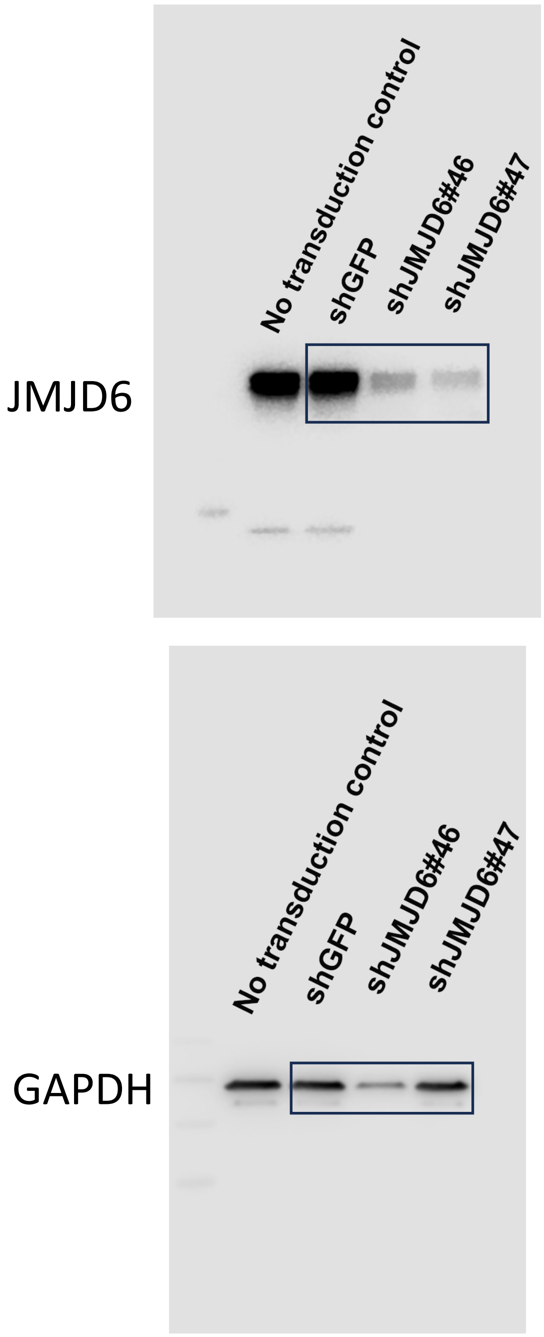

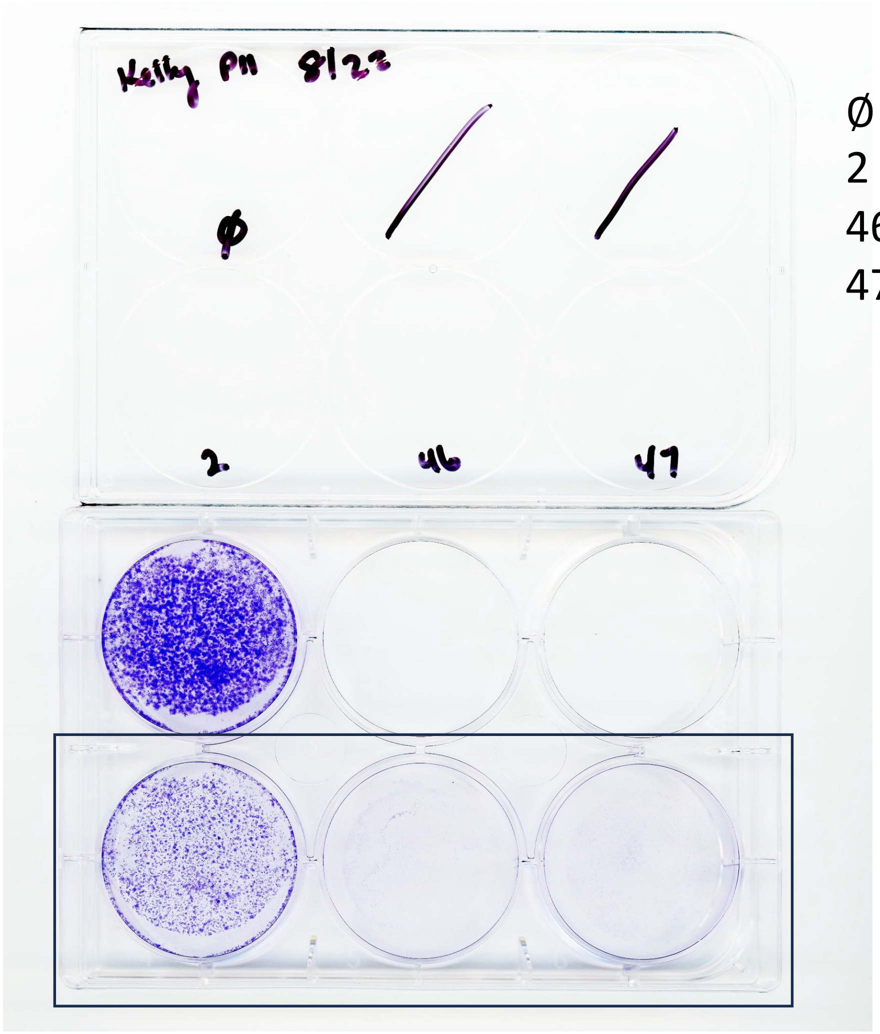

Ø = non transduction control
2 = shGFP
46 = shJMJD6#46
47 = shJMJD6#47

Figure 2C- SIMA

JMJD6

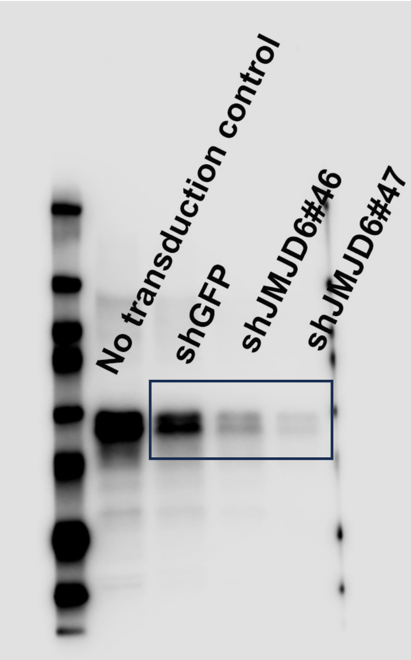

GAPDH

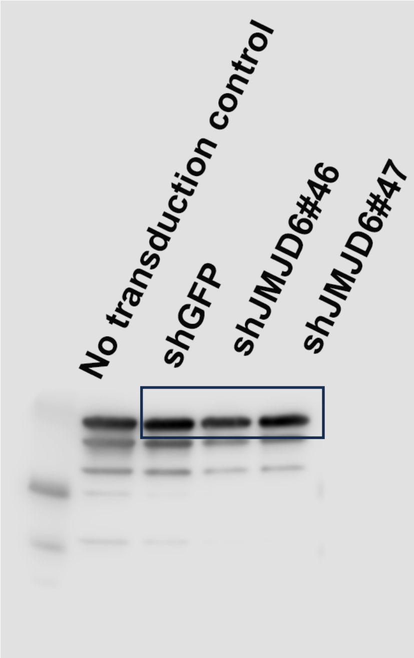

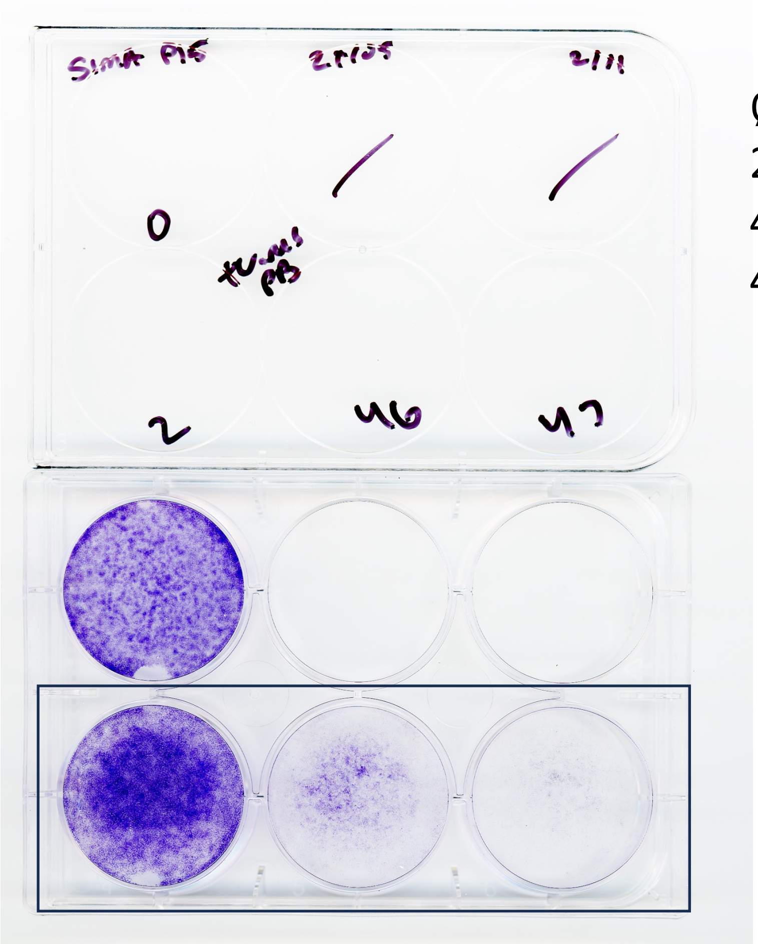

Ø = non transduction control
2 = shGFP
46 = shJMJD6#46
47 = shJMJD6#47

Figure 2C- IMR32 and CHLA20

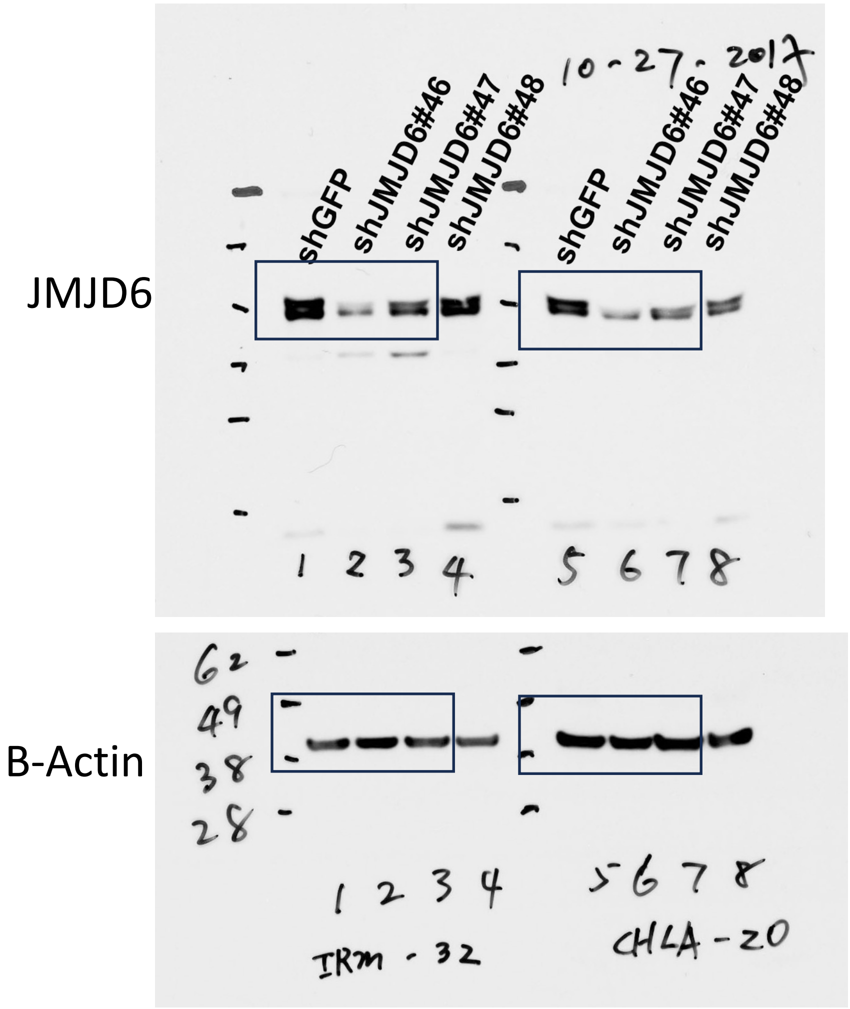

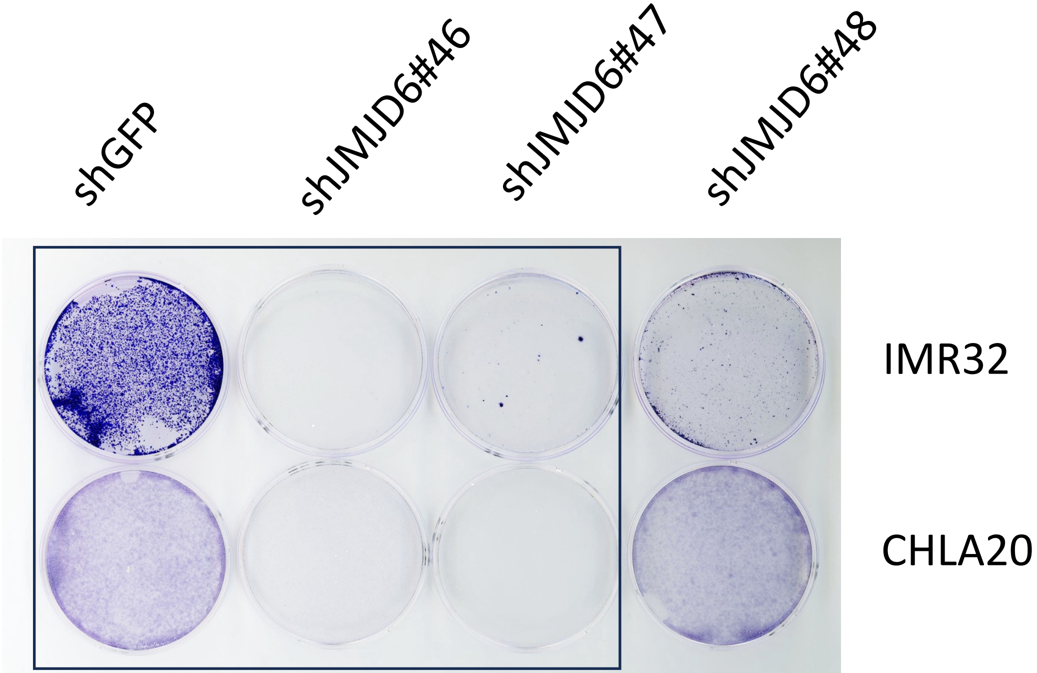

Figure 2F

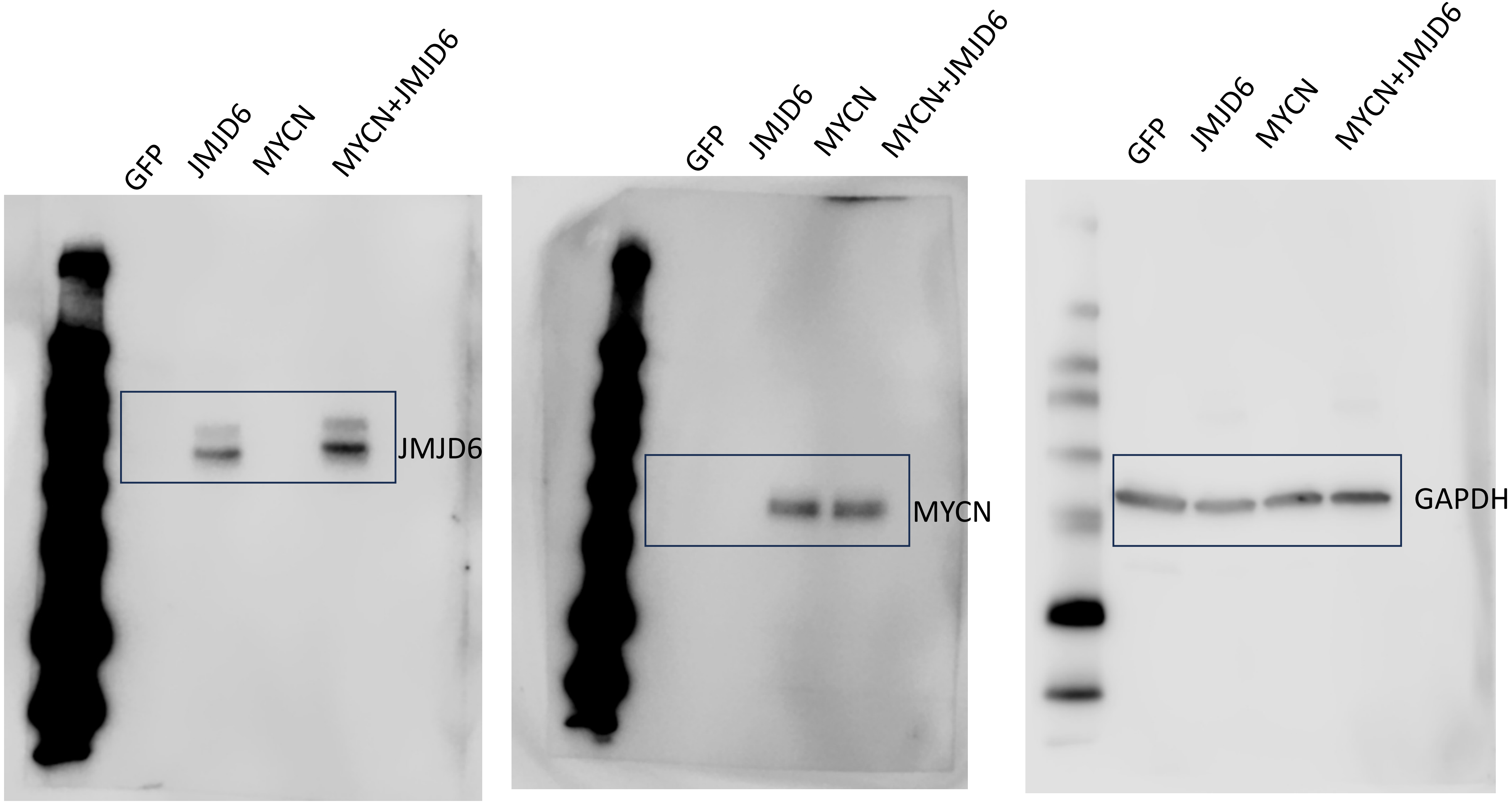
GFP
JMJD6
MYCN
MYCN+JMJD6
JMJD6
GFP
JMJD6
MYCN
MYCN+JMJD6
MYCN
GFP
JMJD6
MYCN
MYCN+JMJD6
GAPDH
