# Supplementary material for: Metabolic reprogramming of cancer cells by JMJD6-mediated pre-mRNA splicing associated with therapeutic response to splicing inhibitor
Source: eLife. 2024 Mar 15;12:RP90993. doi: 10.7554/eLife.90993 (PMC10942784; doi:10.7554/eLife.90993)

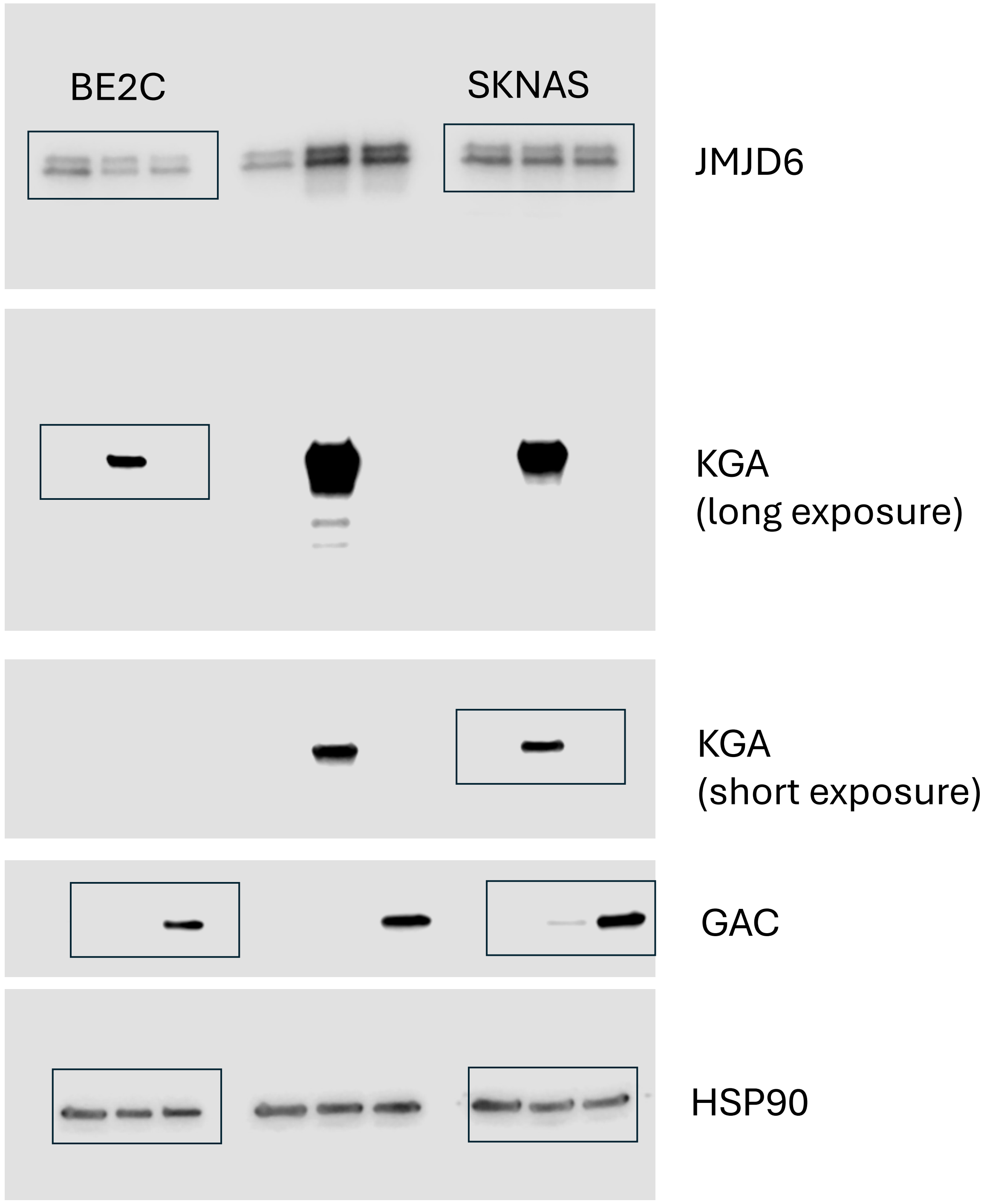

Figure 5A

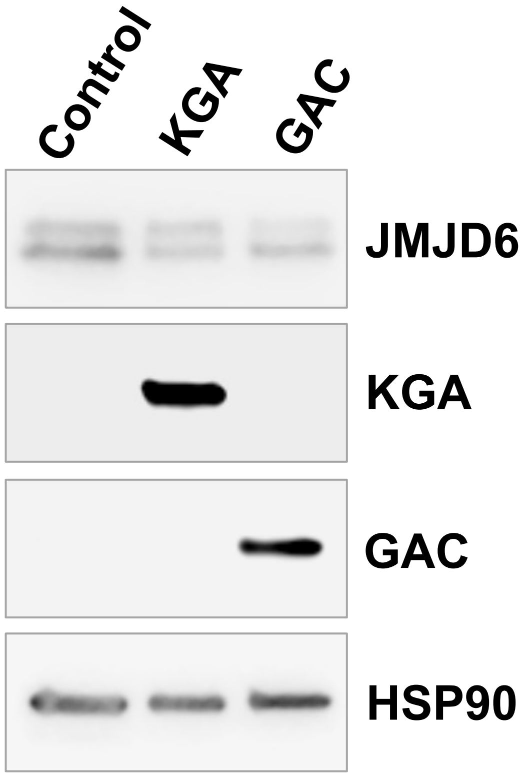

Figure 5C

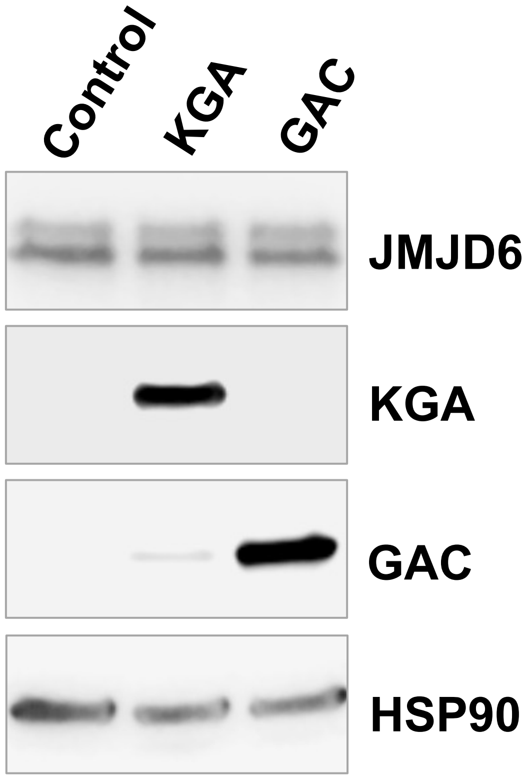

Figure 5B

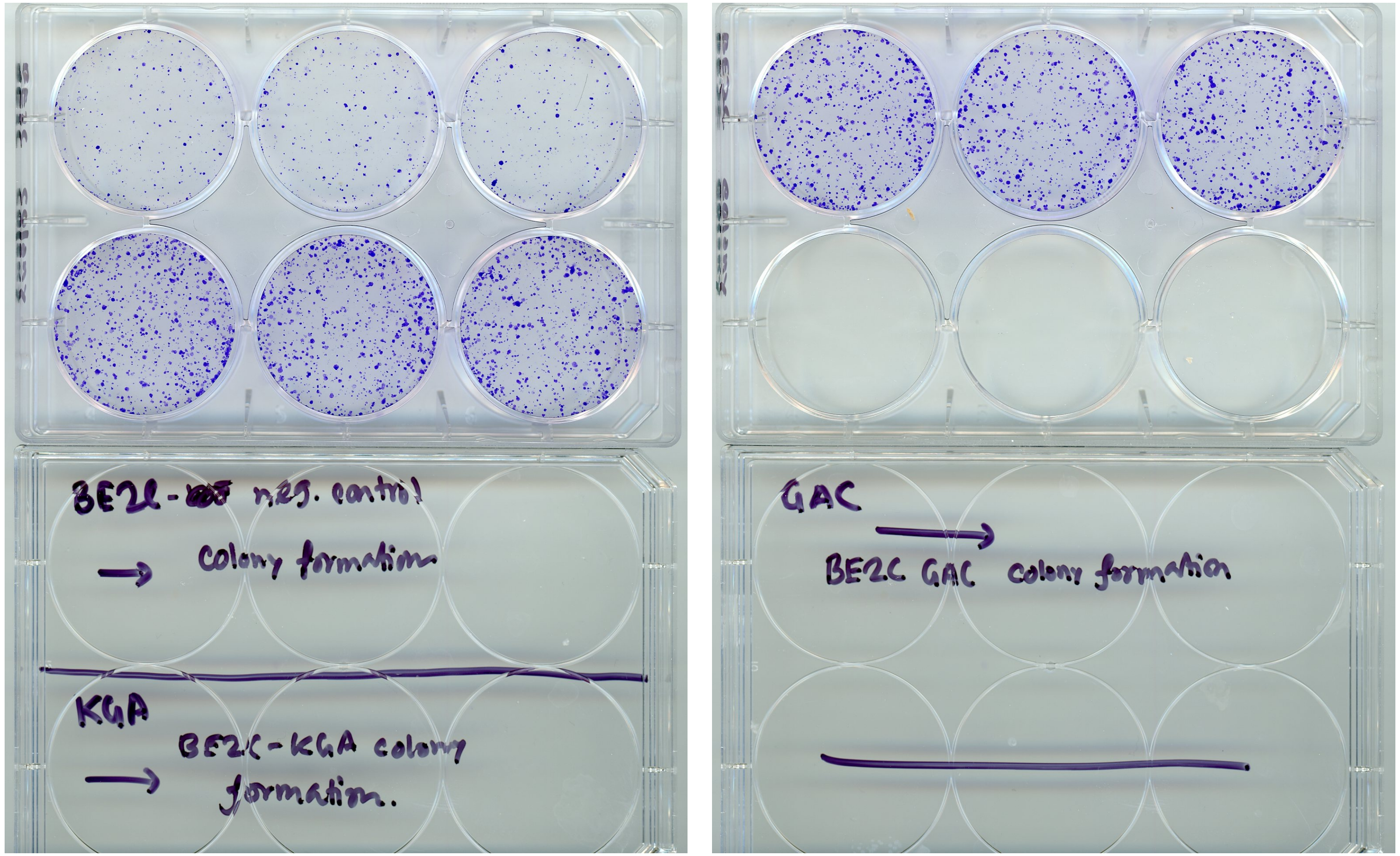
BE2C- neg. control
colony formation
KGA
BE2C-KGA colony formation.
GAC
BE2C GAC colony formation

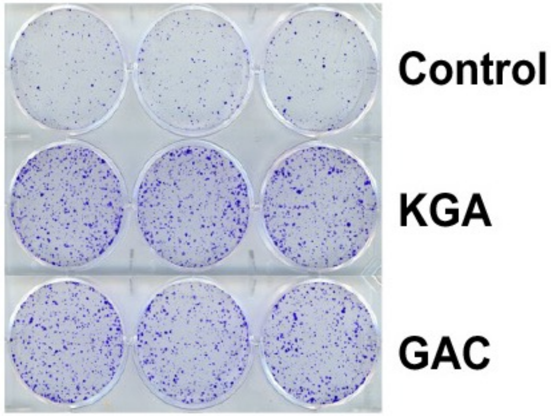
Control
KGA
GAC

Figure 5D

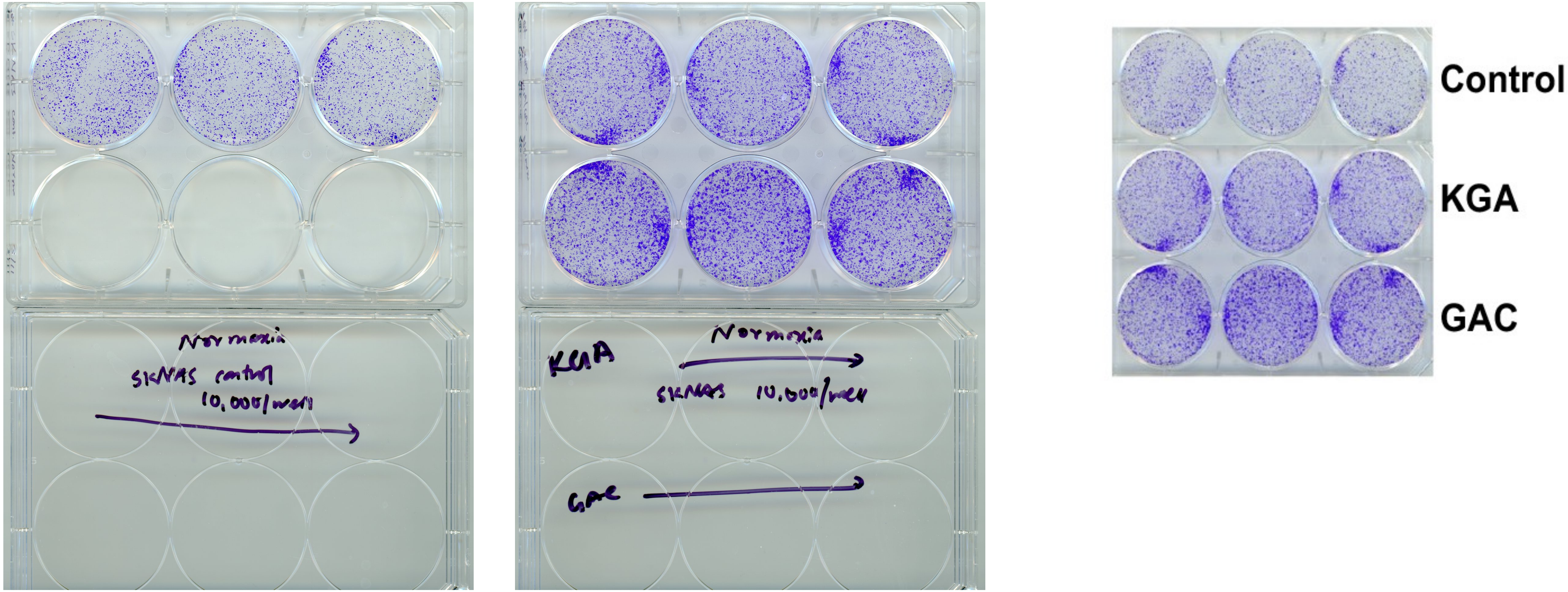
Normoxia
SKNAS control
10,000/well
KGA
Normoxia
SKNAS 10,000/well
GAC
Control
KGA
GAC

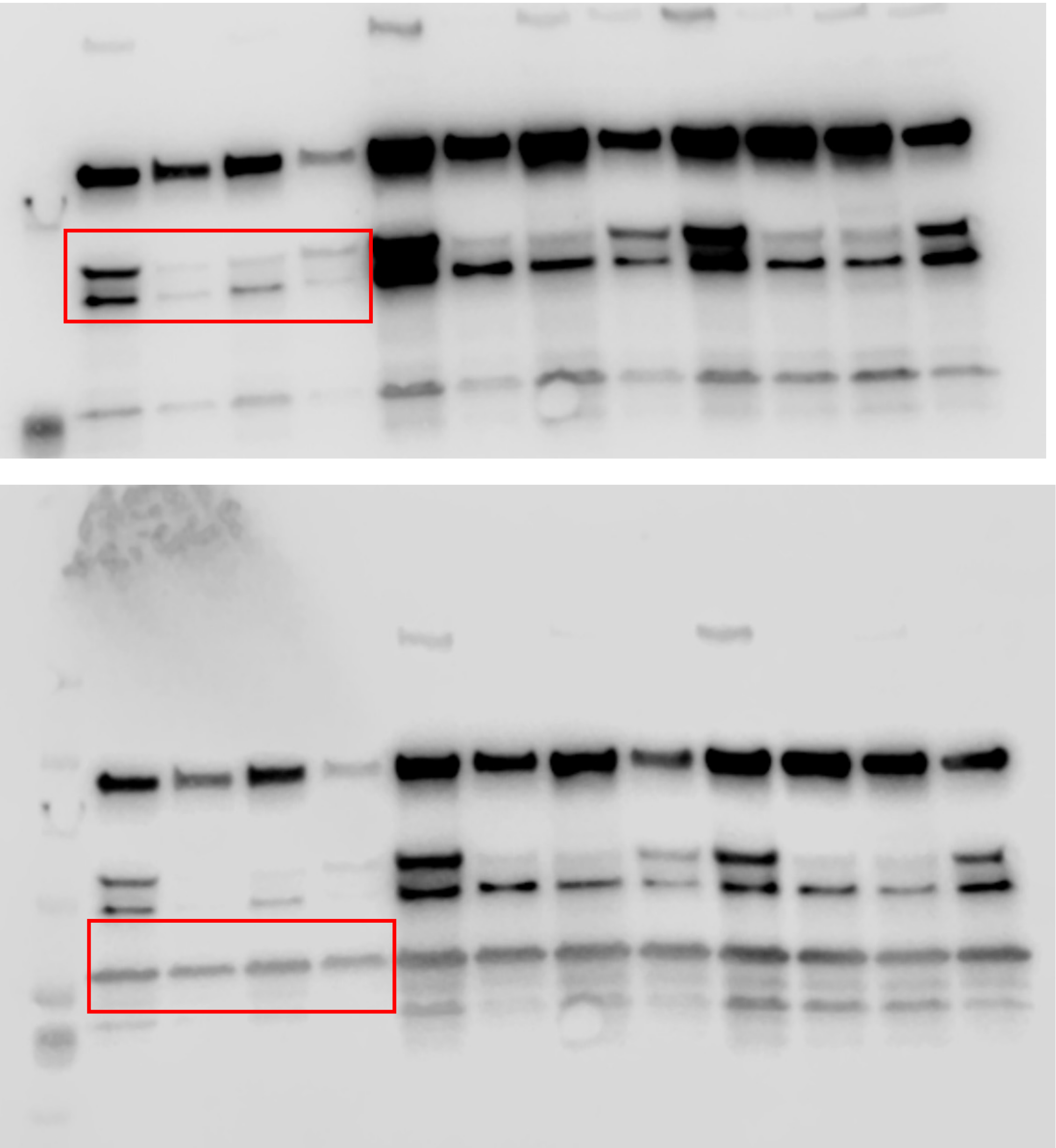

Figure 5G

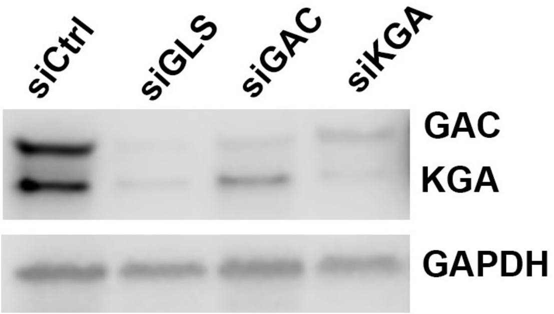

Figure 5H

BE2C
siCtrl
siGLS
siGAC
siKGA

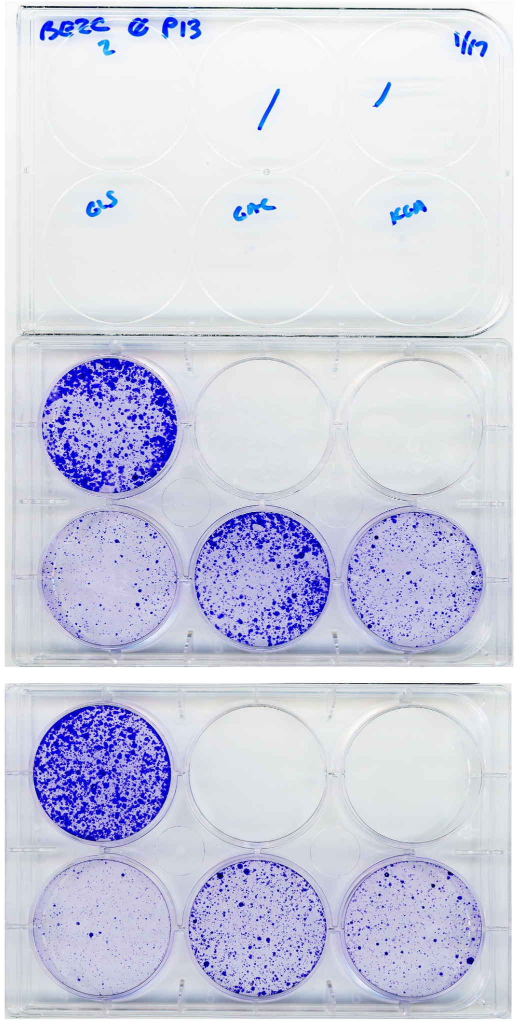

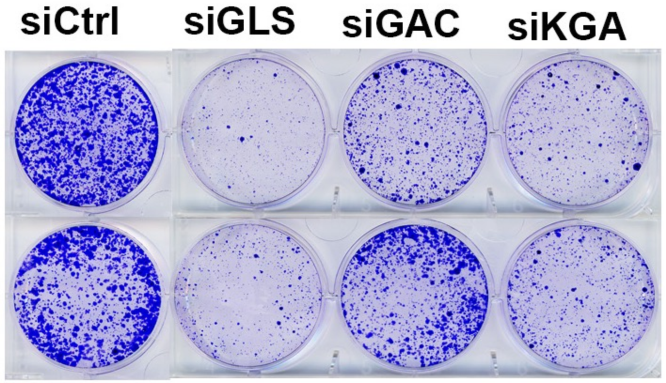

Supplement: Source data 1. [file elife-90993-data1.zip › Figure 5-data source.pdf]
