# Supplementary figures and images for: Metabolic reprogramming of cancer cells by JMJD6-mediated pre-mRNA splicing associated with therapeutic response to splicing inhibitor
Source: eLife. 2024 Mar 15;12:RP90993. doi: 10.7554/eLife.90993 (PMC10942784; doi:10.7554/eLife.90993)

Figure 2—Supplement 2A, B

A


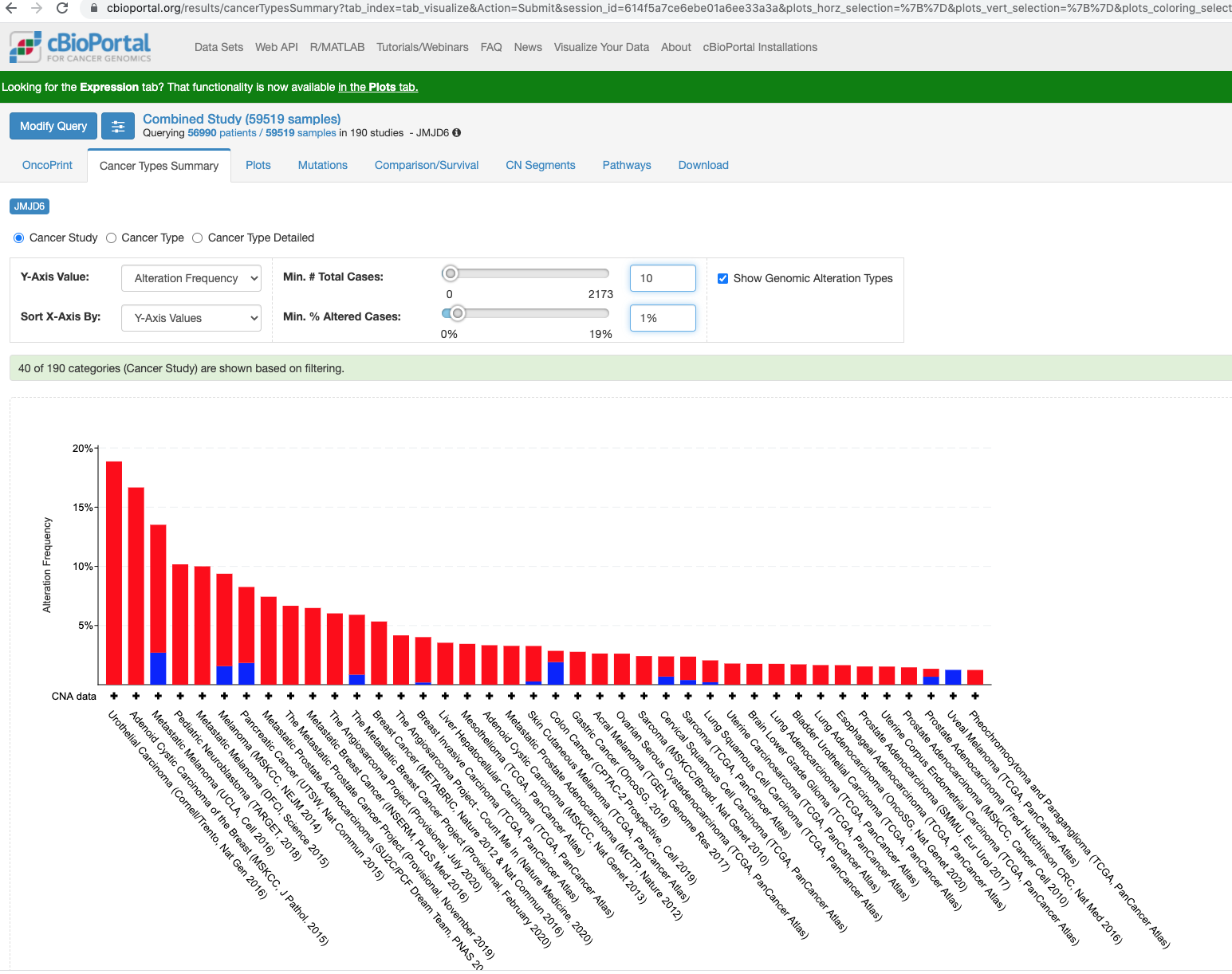


B


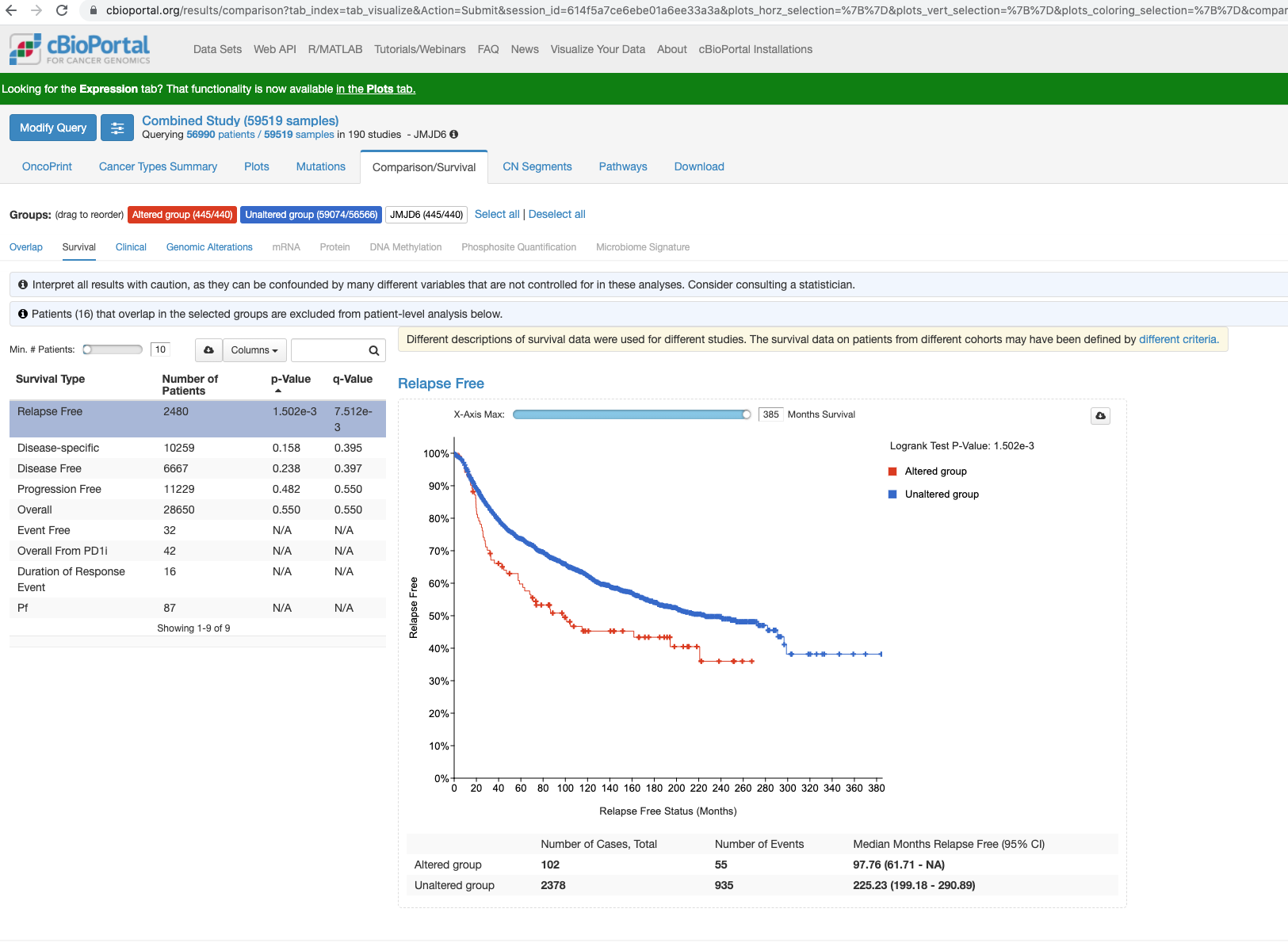

Supplement: Source data 1. [file elife-90993-data1.zip › Figure 2-Supplement 2-data source.docx]

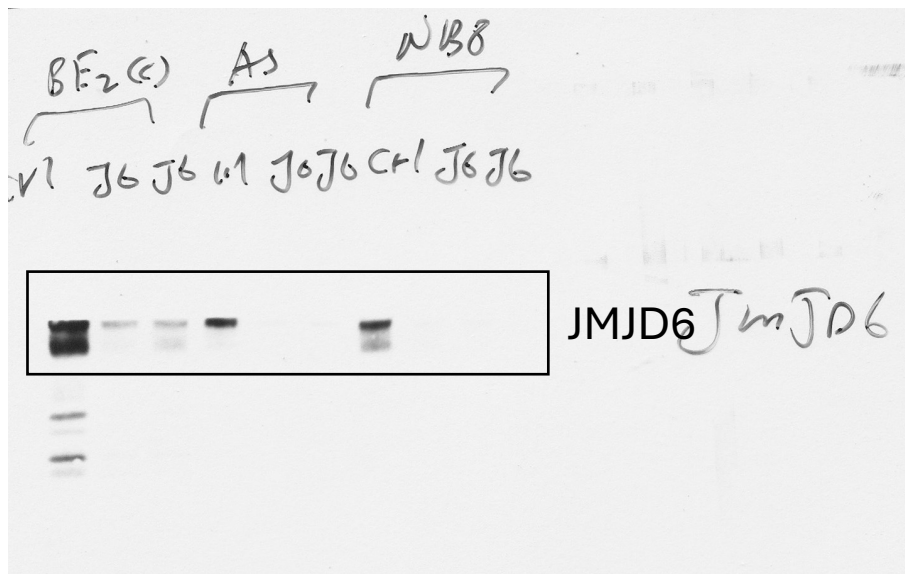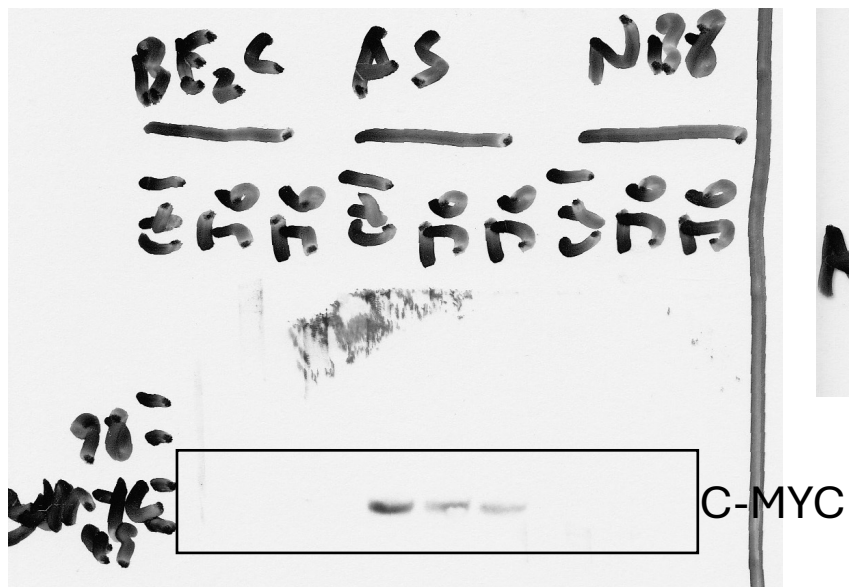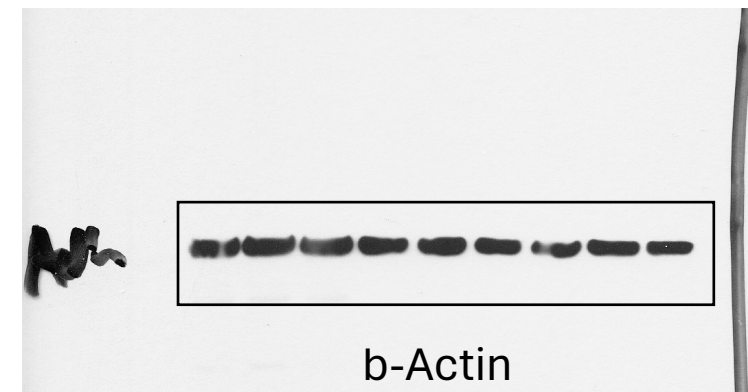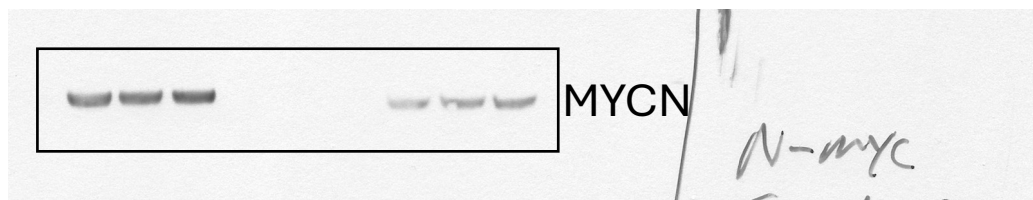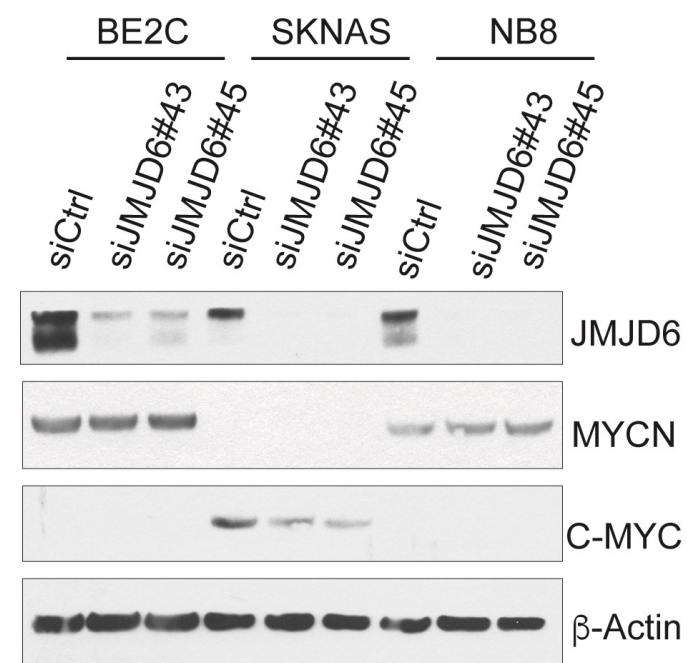

Supplement: Source data 1. [file elife-90993-data1.zip › Figure 3-supplement 1-data source.pdf]

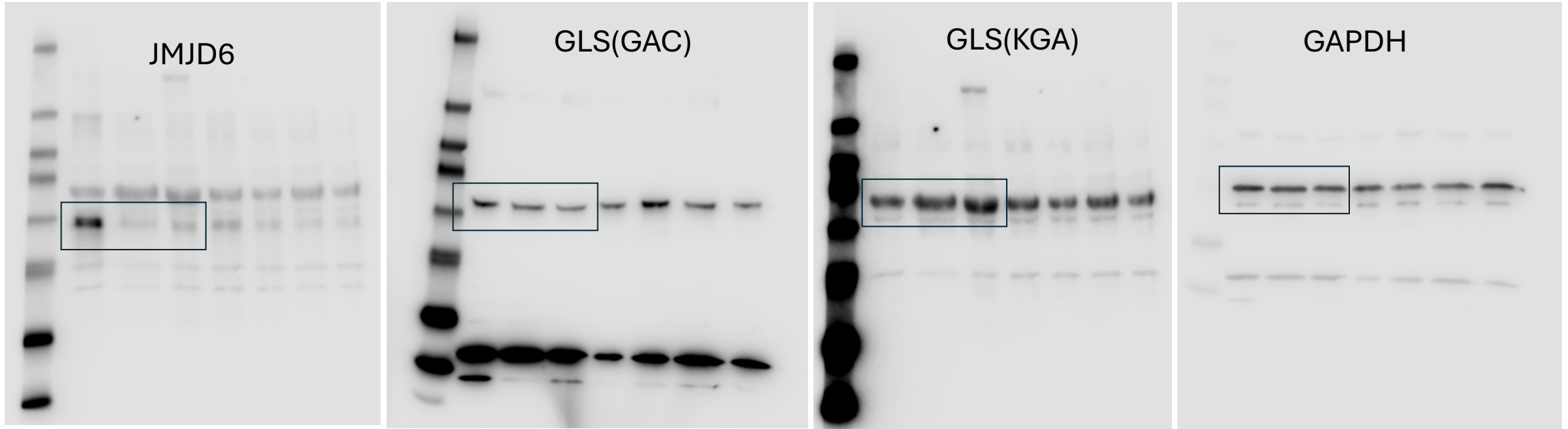

Figure 4C

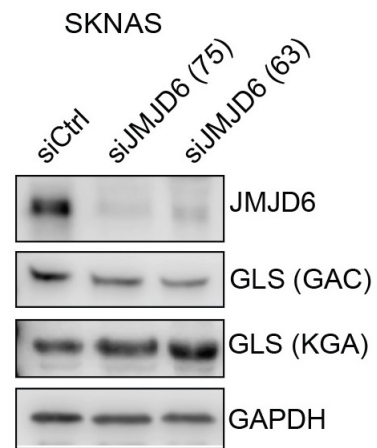

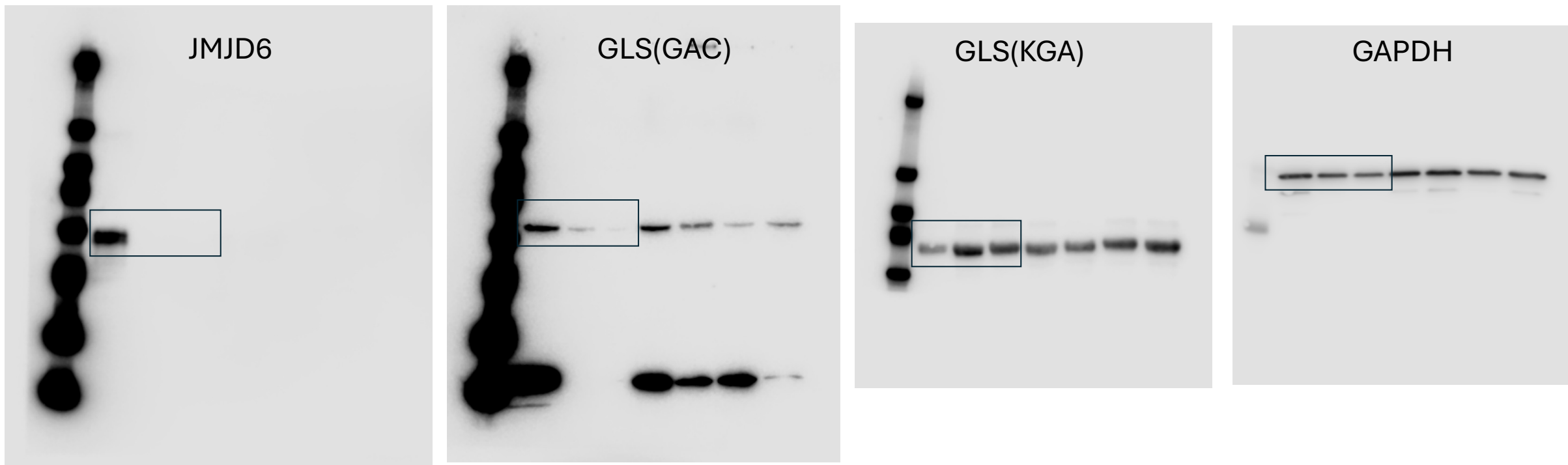

Figure 4C

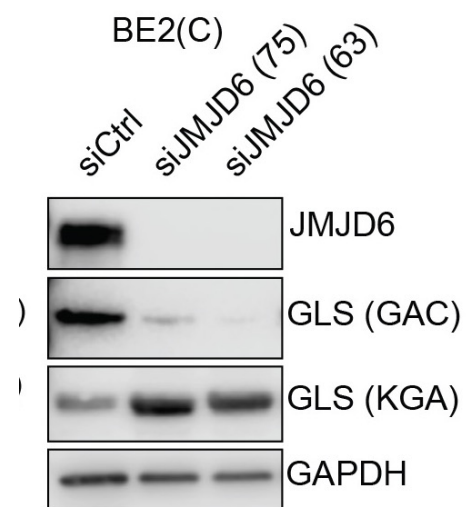

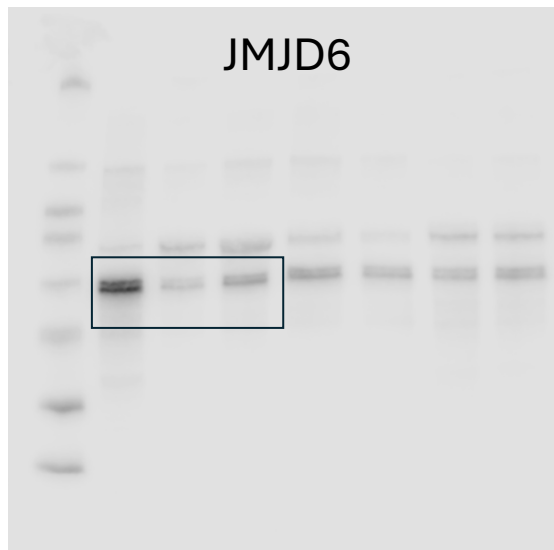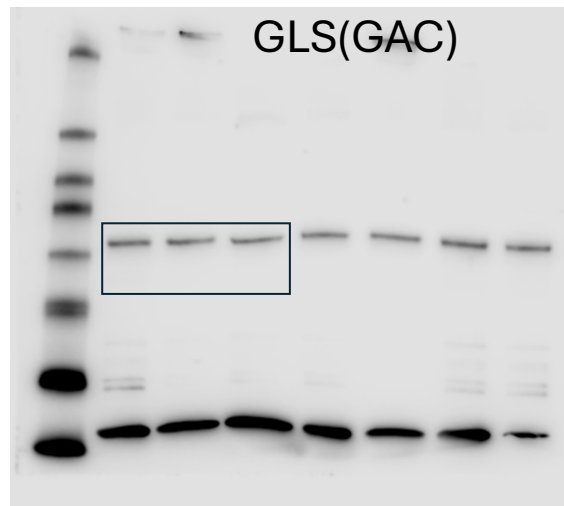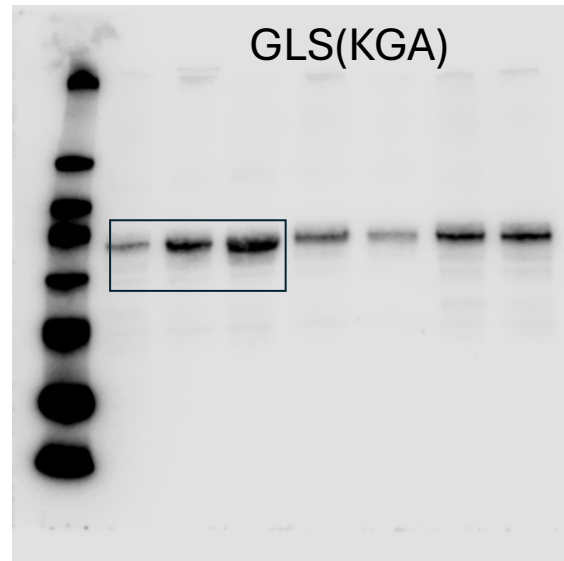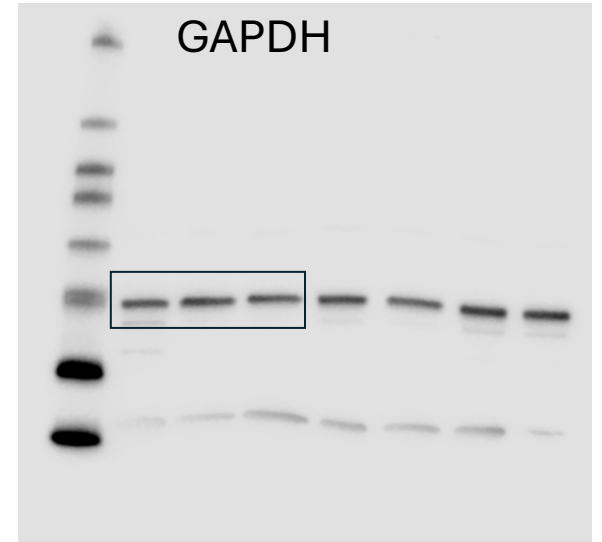

Figure 4C

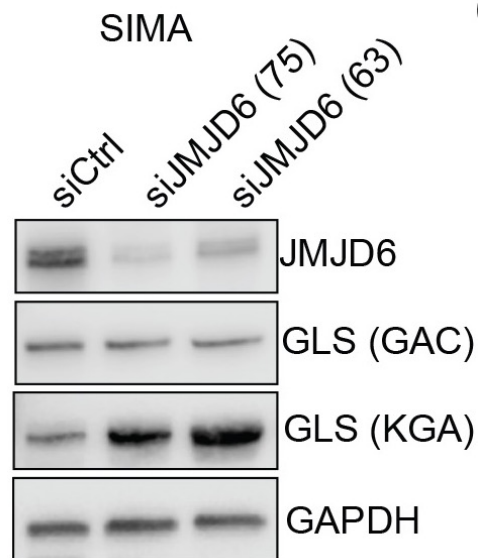

Figure 4E

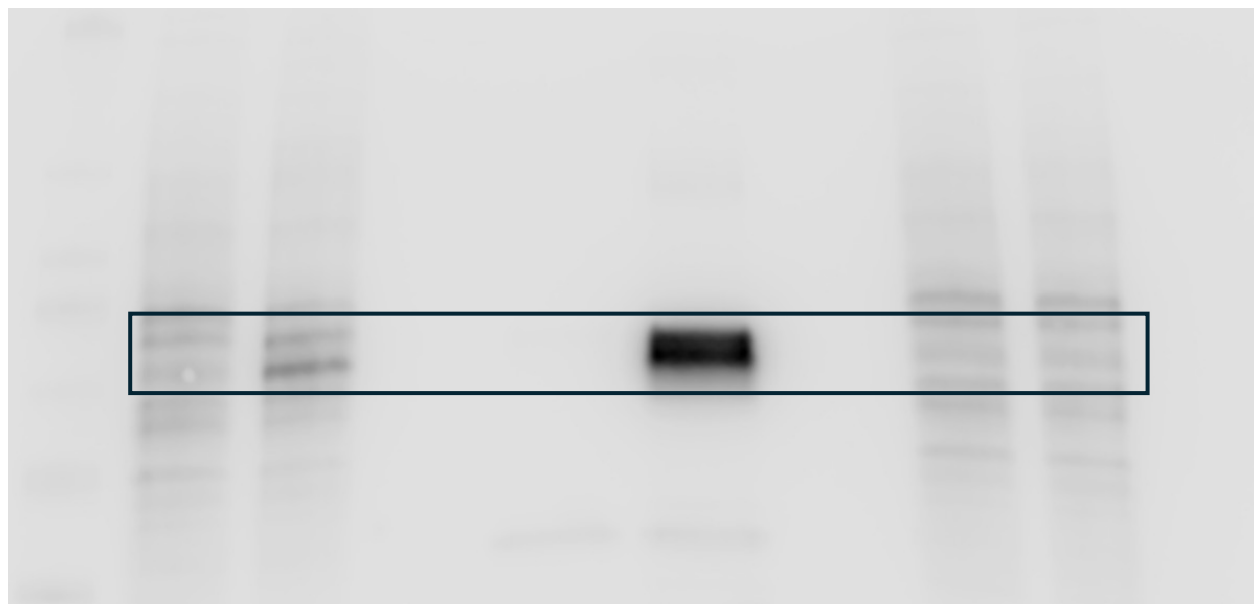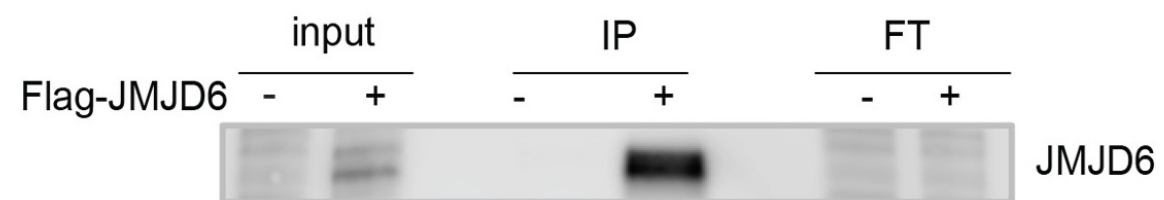

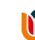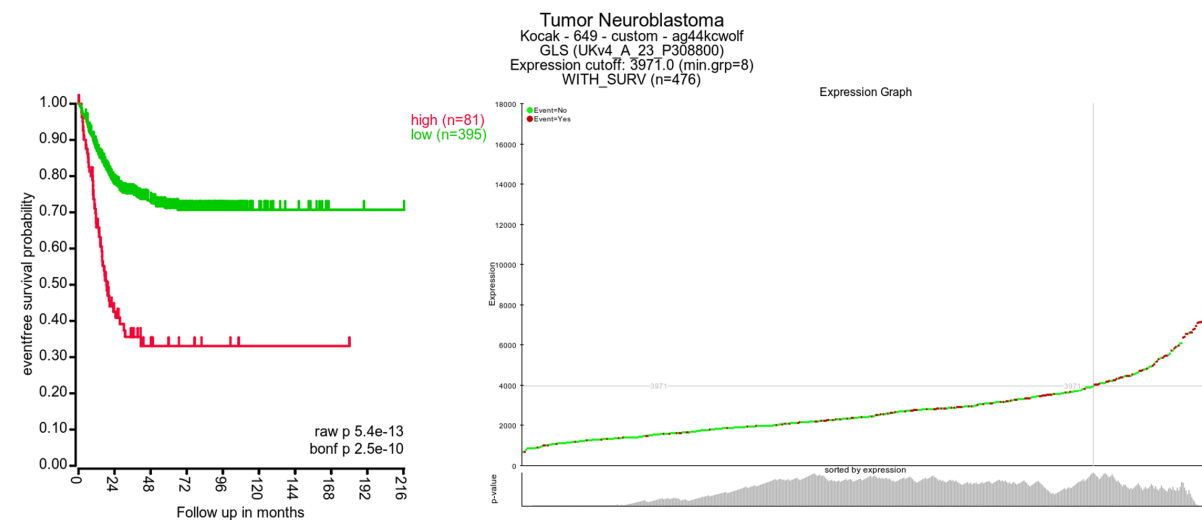

Figure 4F

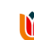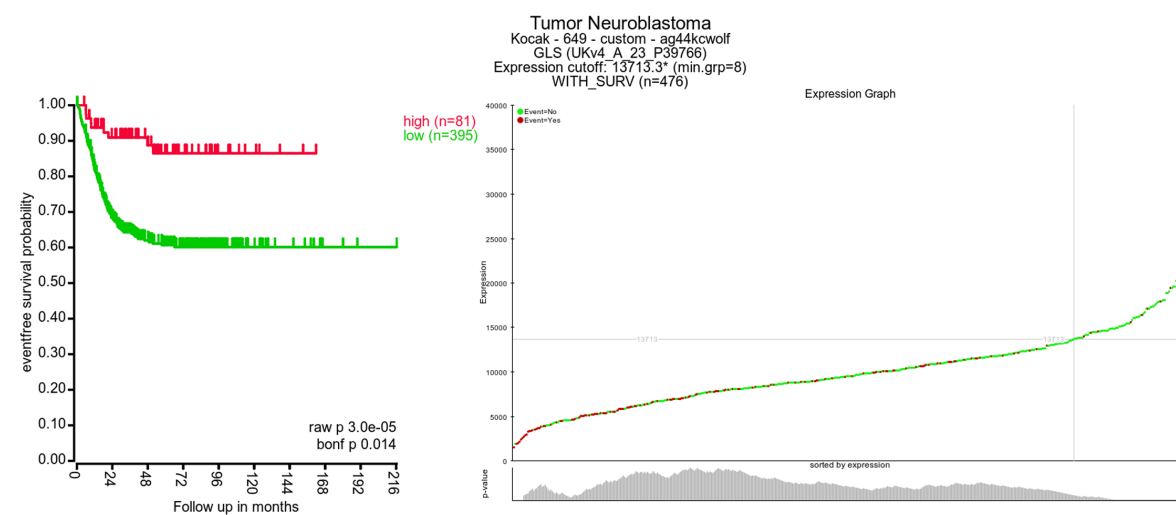

Supplement: Source data 1. [file elife-90993-data1.zip › Figure 4-data source.pdf]

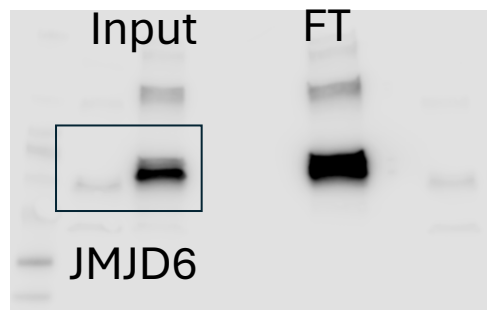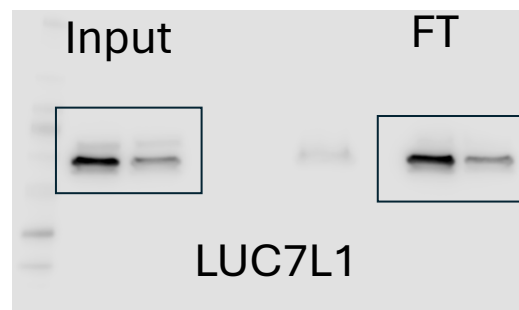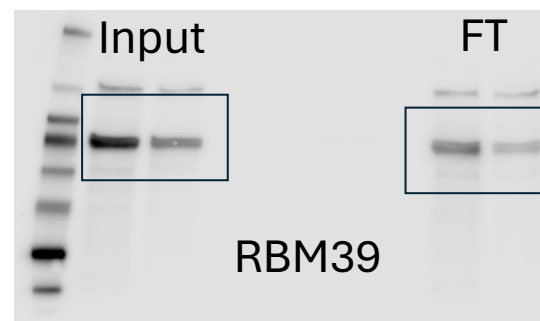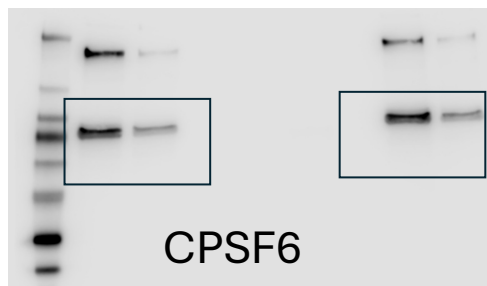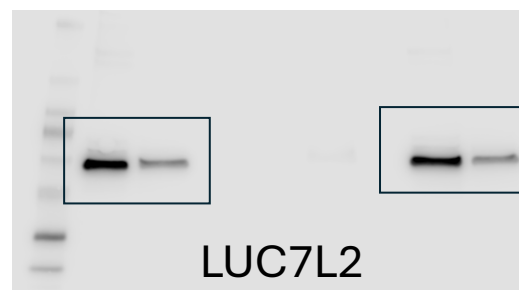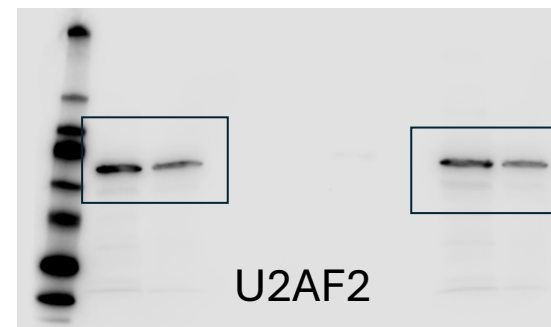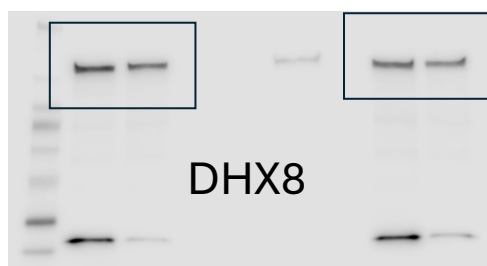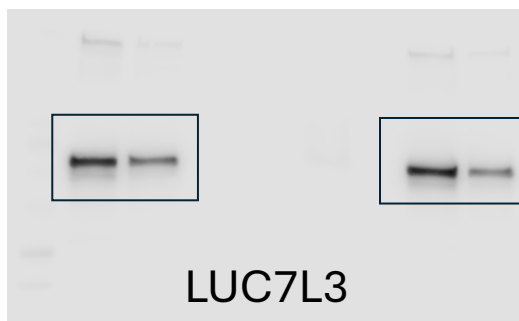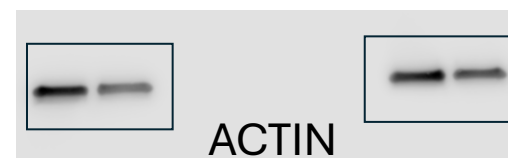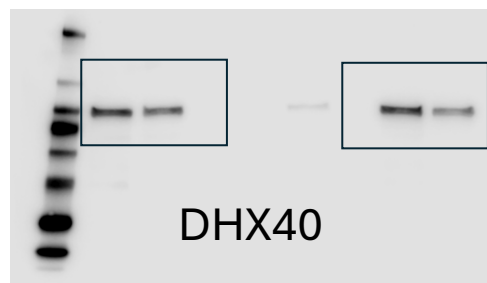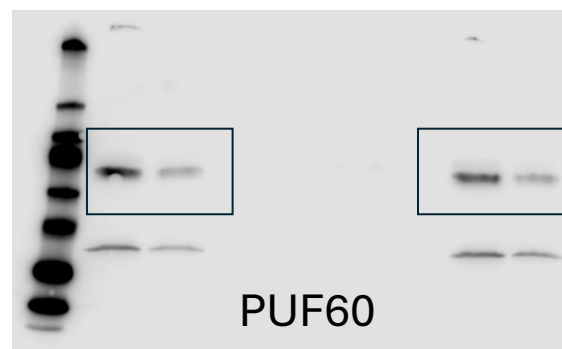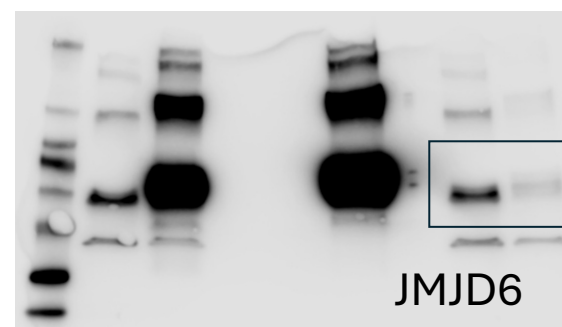

Figure 6B

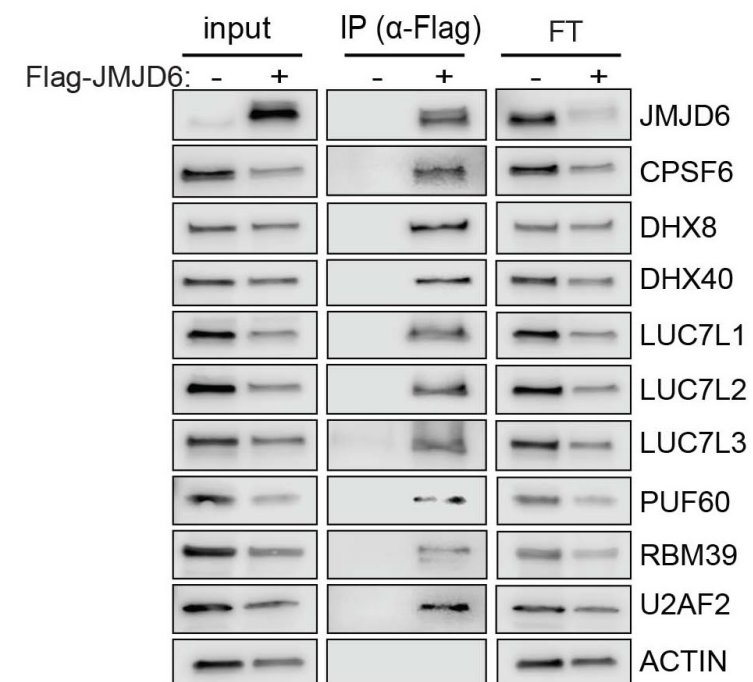

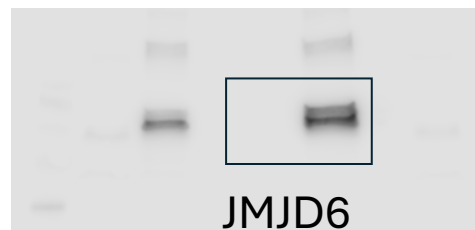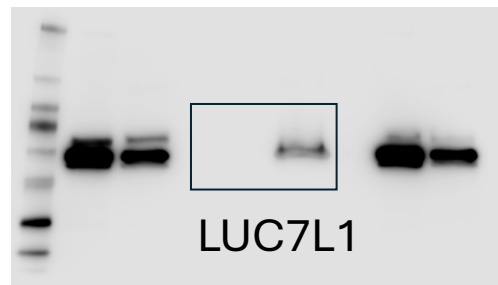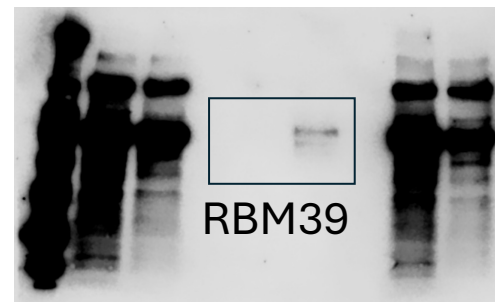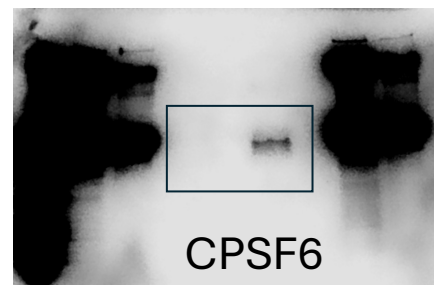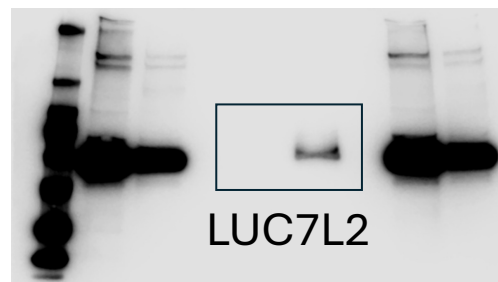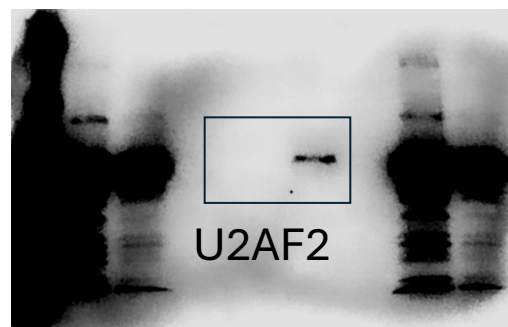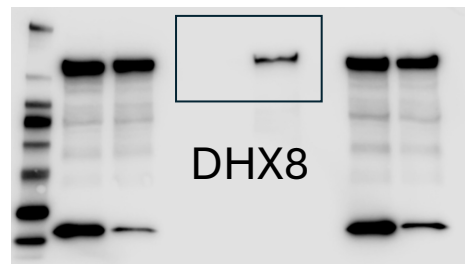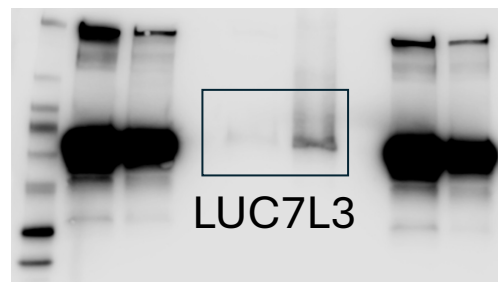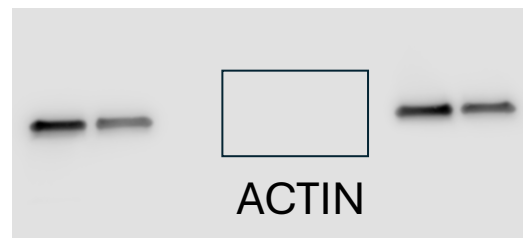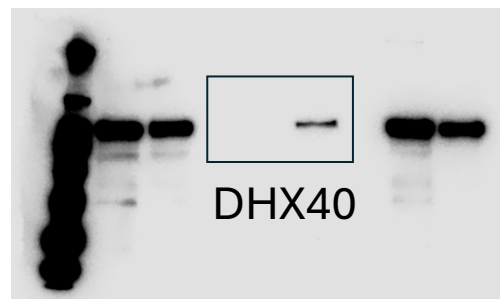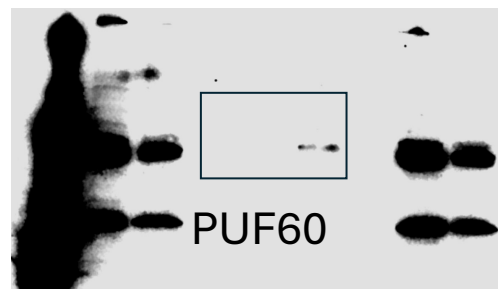

Figure 6B

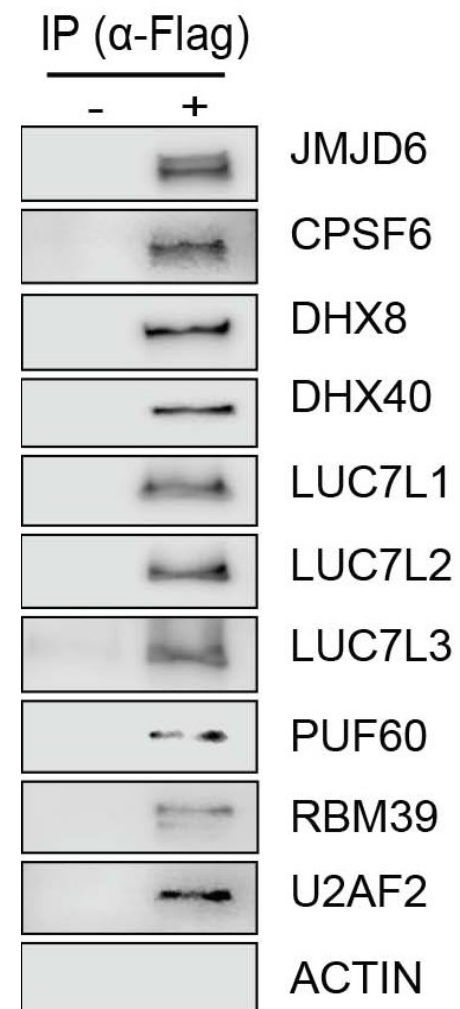

Figure 6C

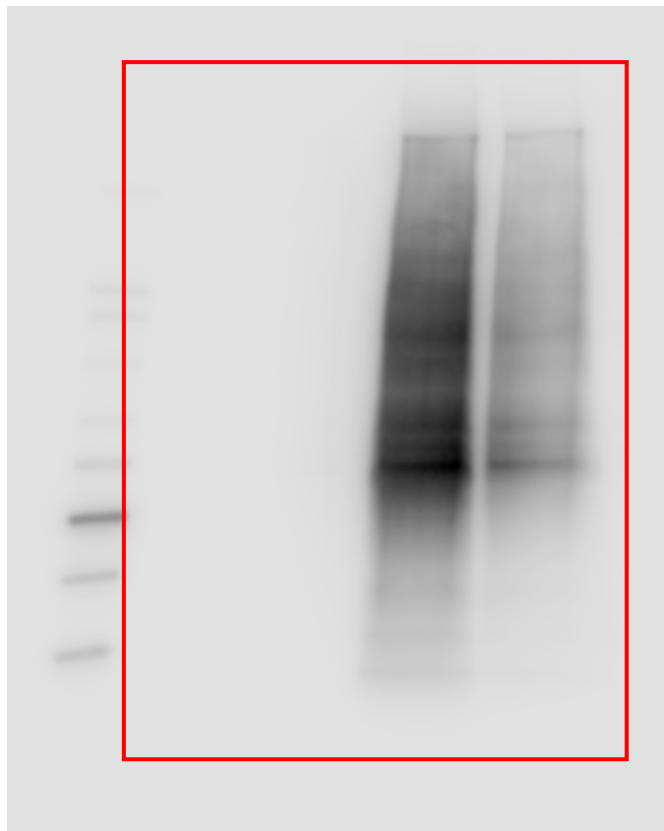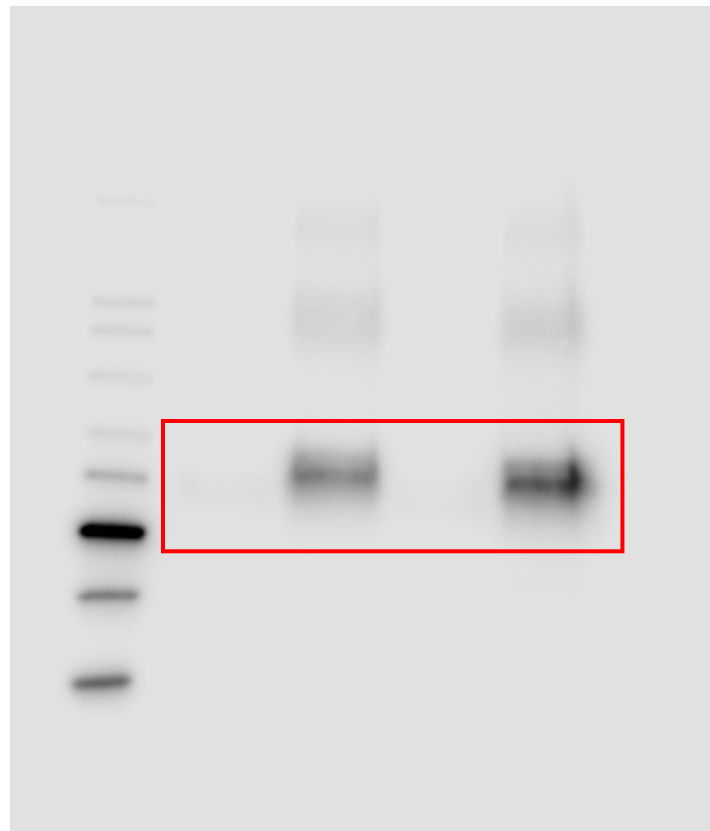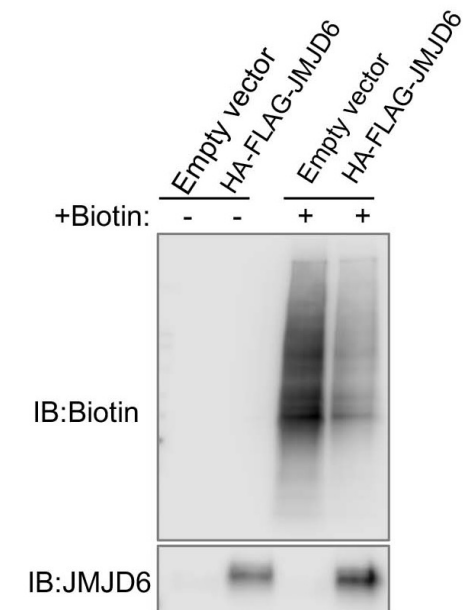

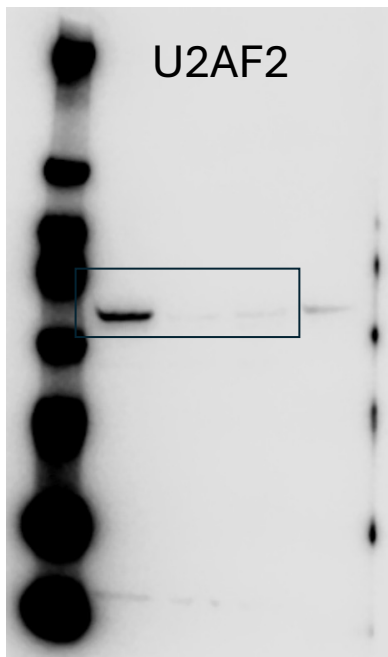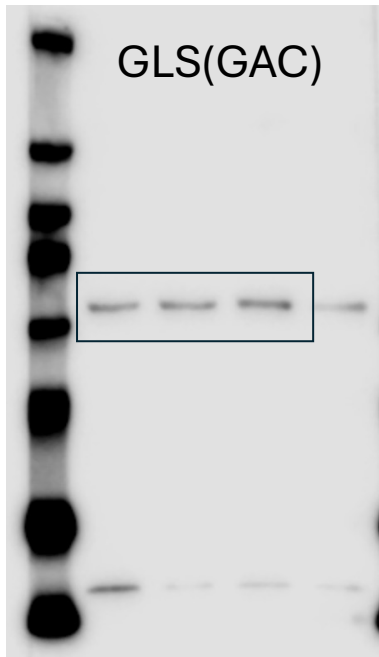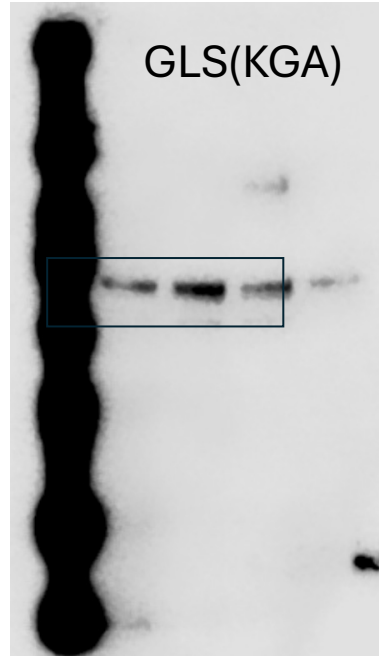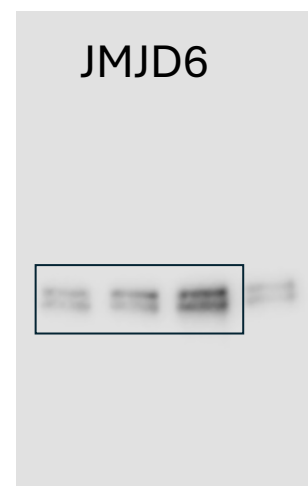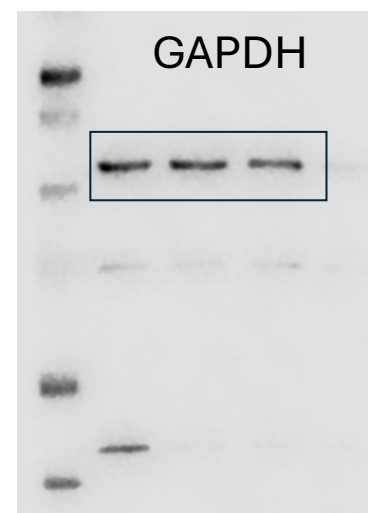

Figure 6D

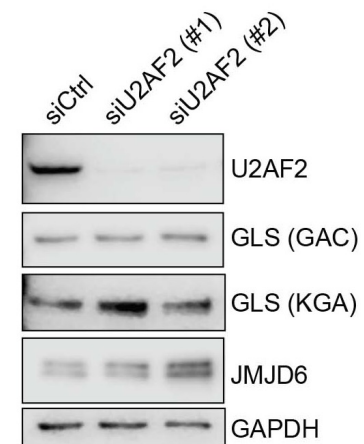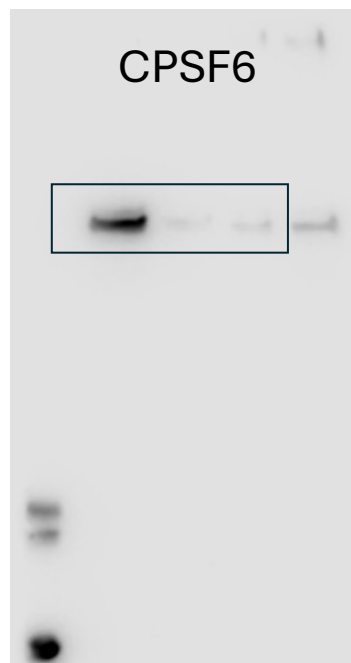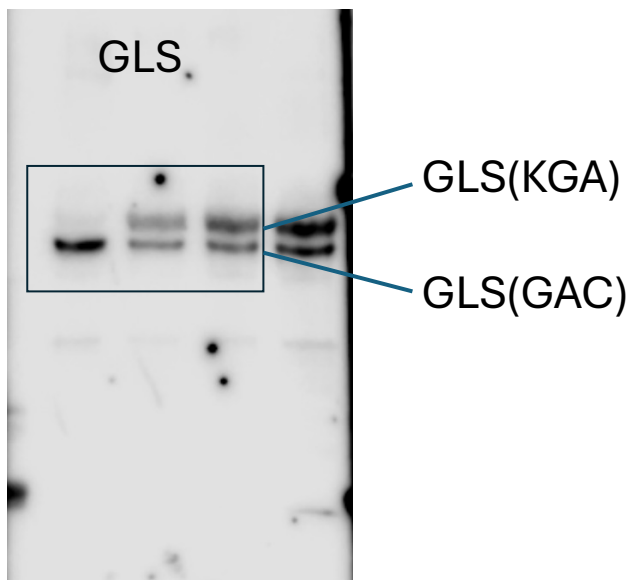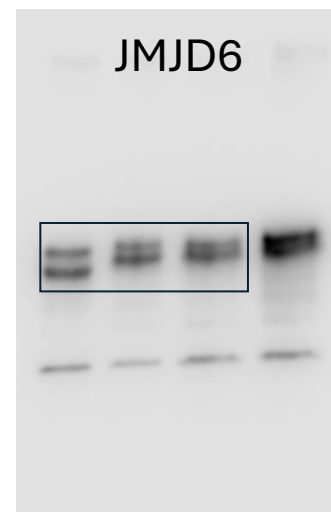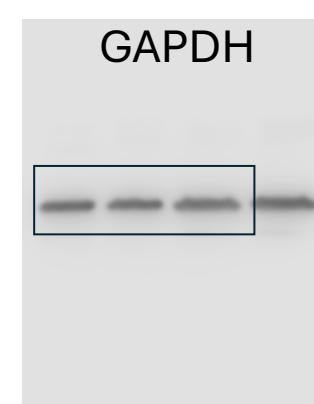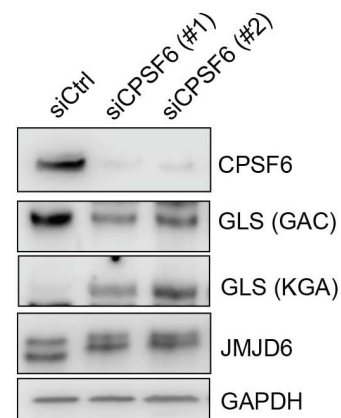

Figure 6E

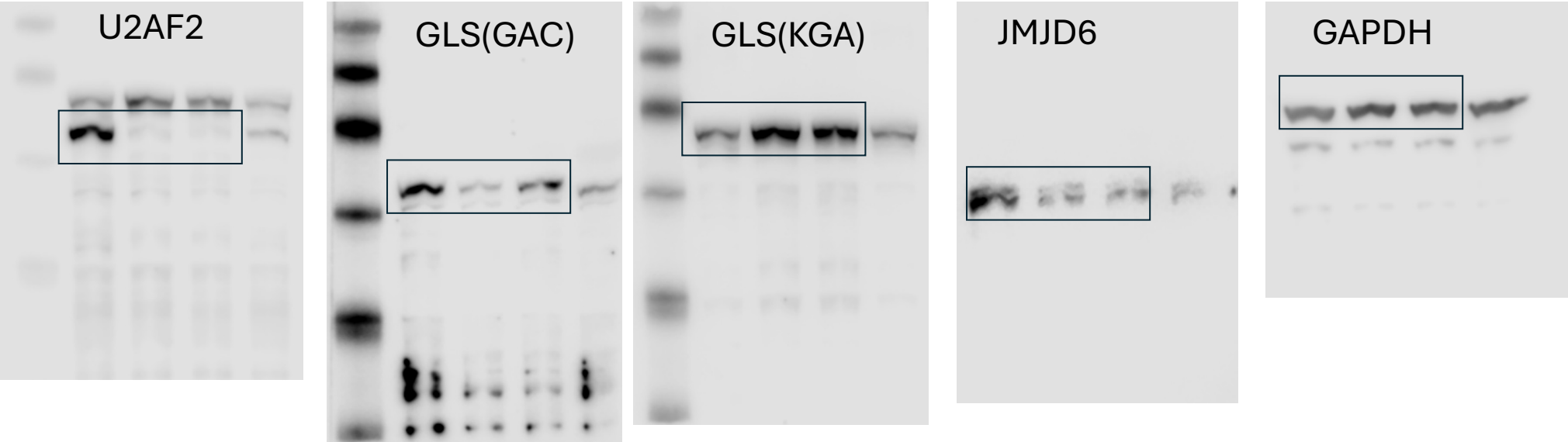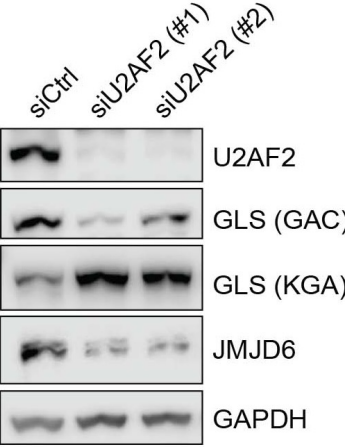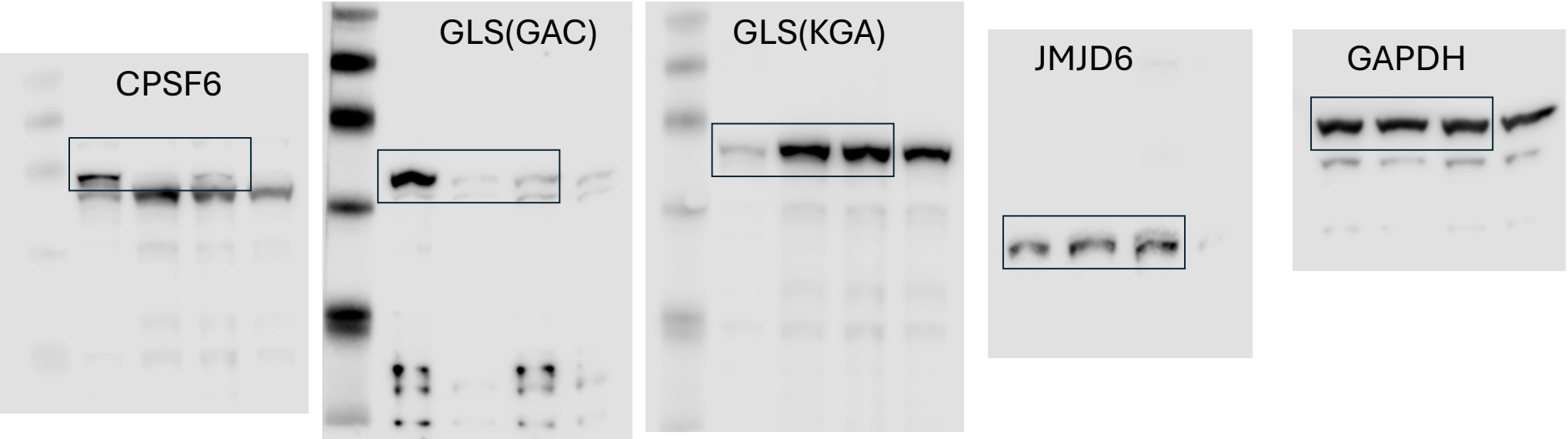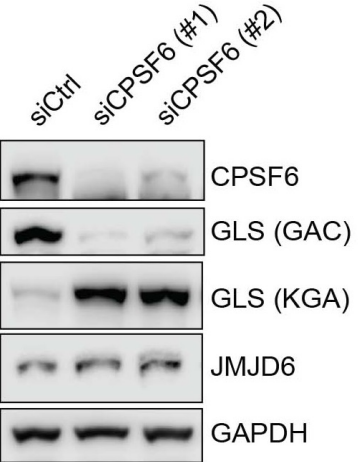

Supplement: Source data 1. [file elife-90993-data1.zip › Figure 6-data source.pdf]

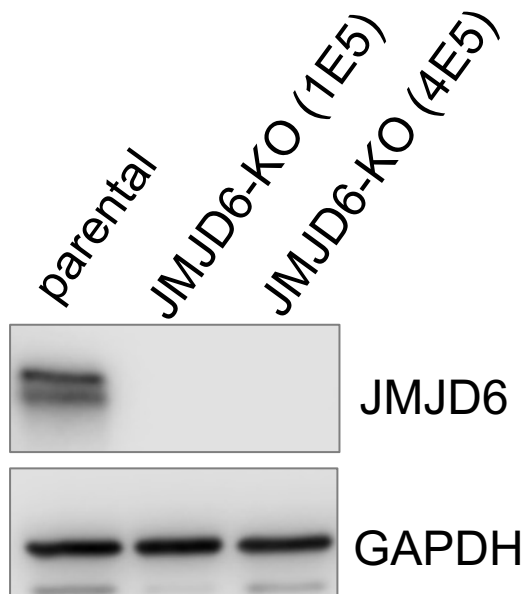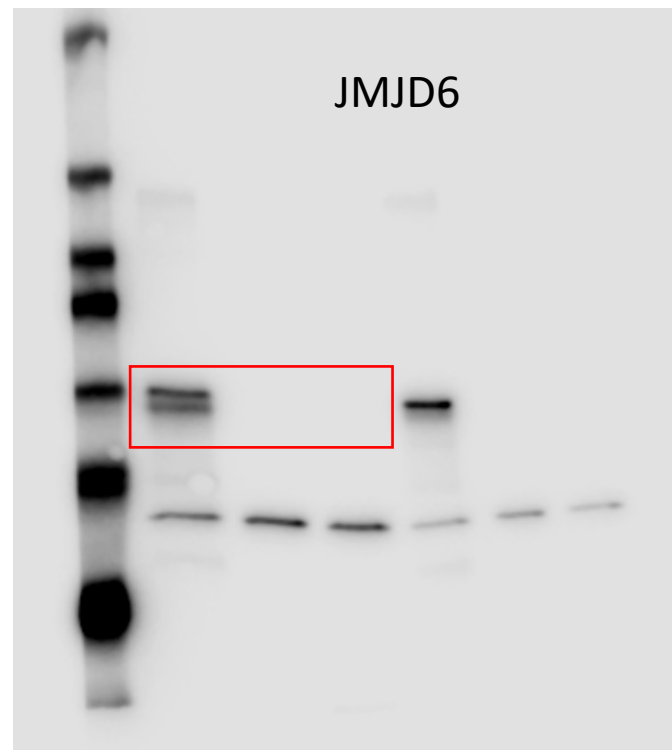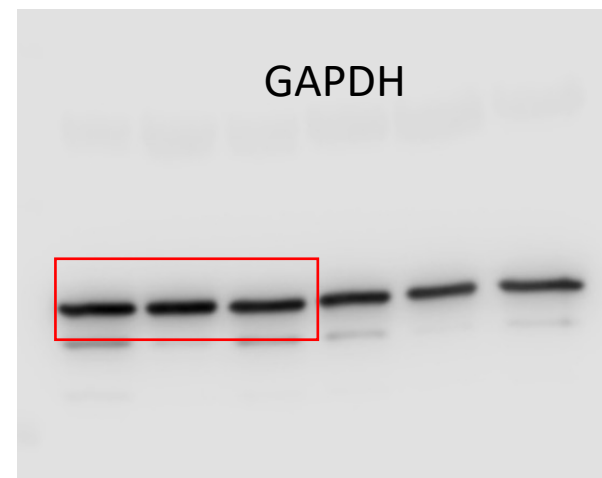

Supplement: Source data 1. [file elife-90993-data1.zip › Figure 7-supplenment 1-data source.pdf]

Figure 8B

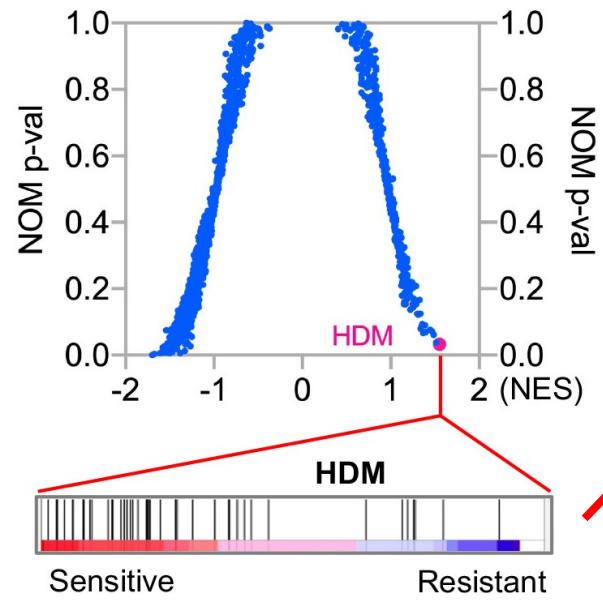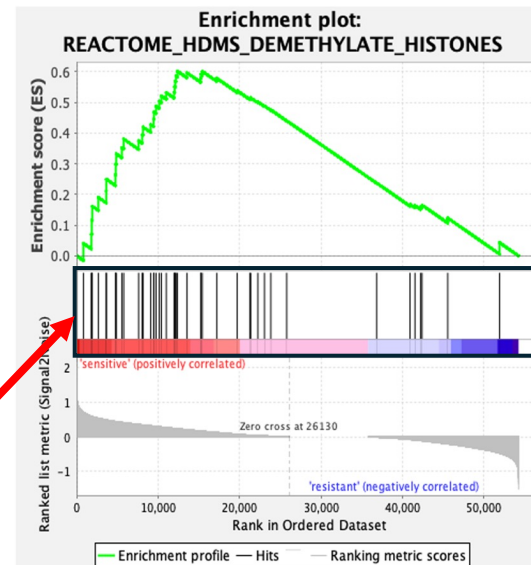

Figure 8C

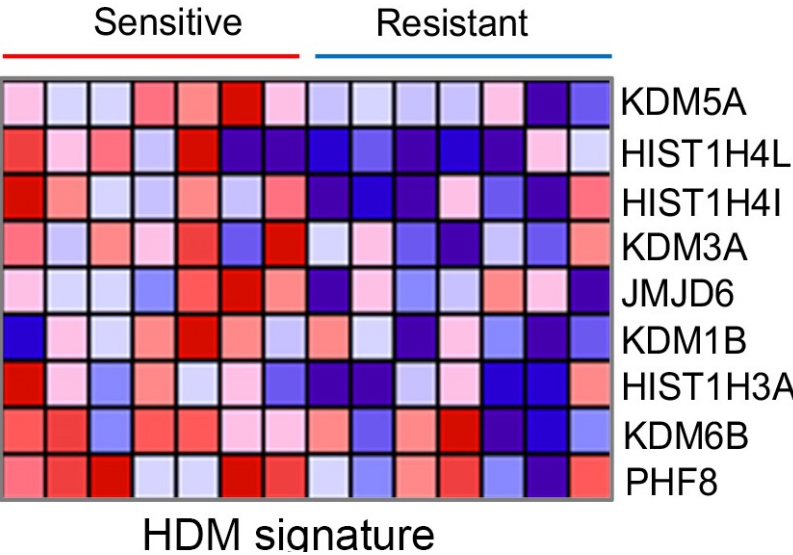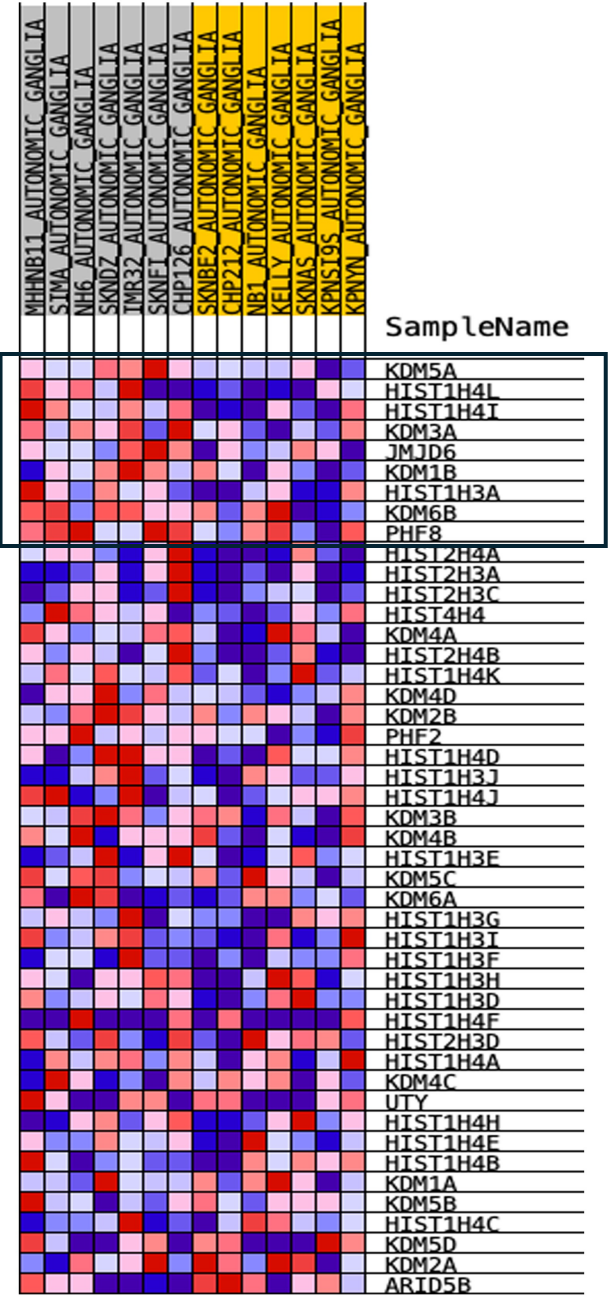

Figure 8E

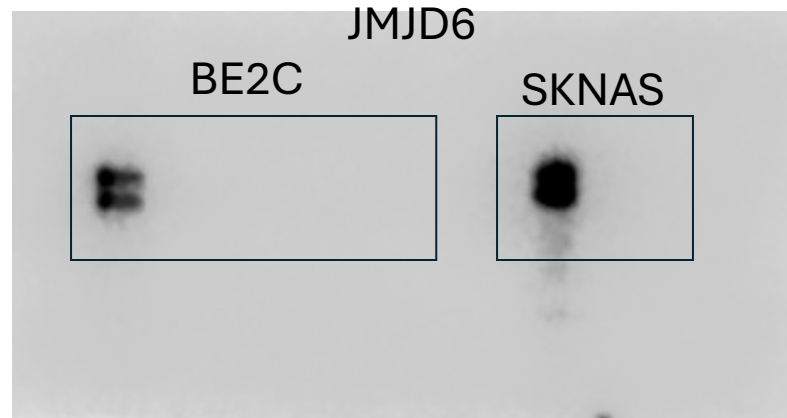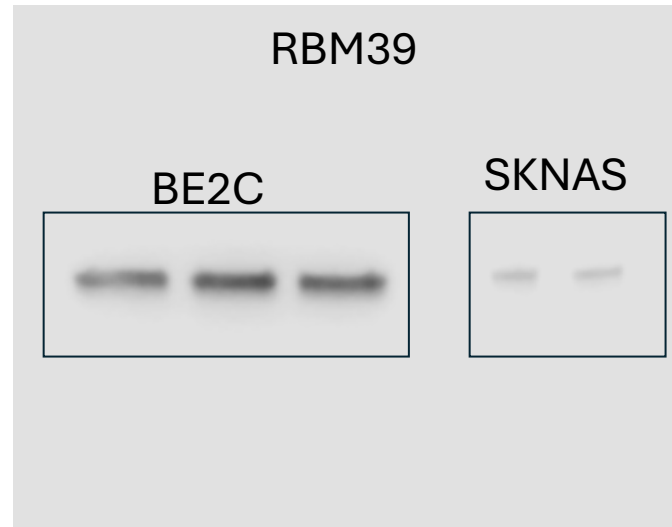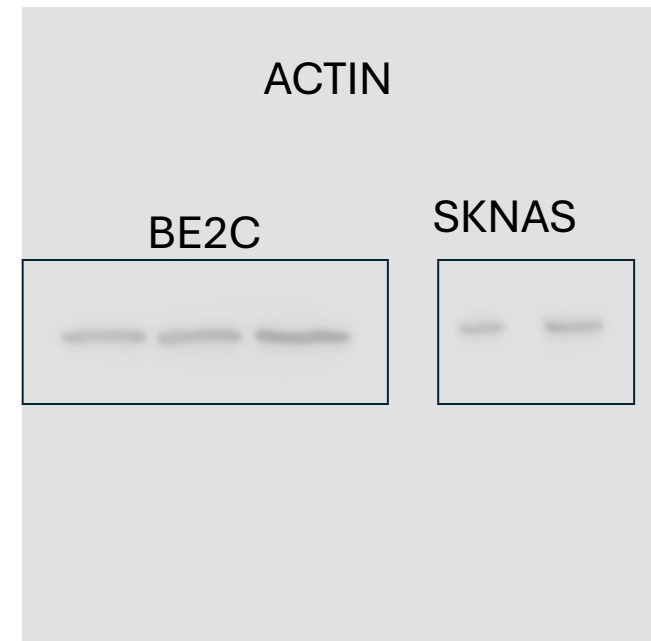

Figure 8E

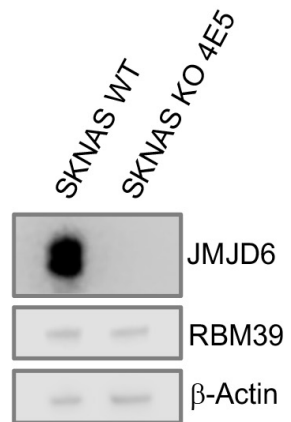

Figure 8H

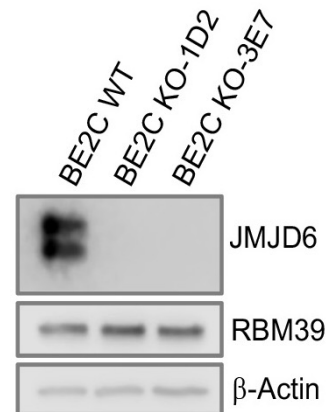

Figure 8F

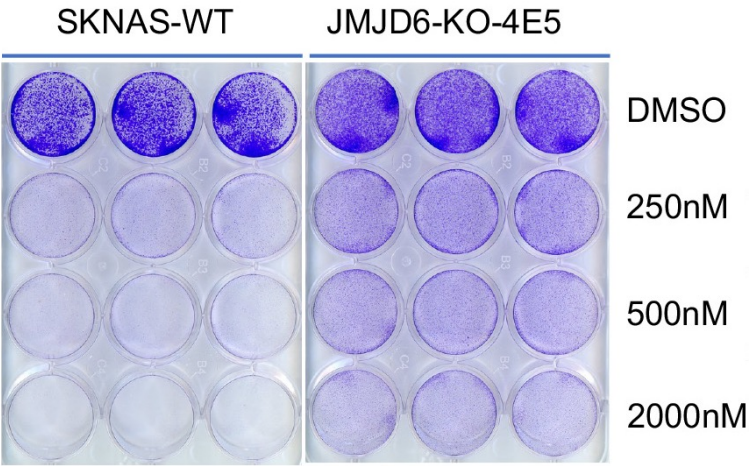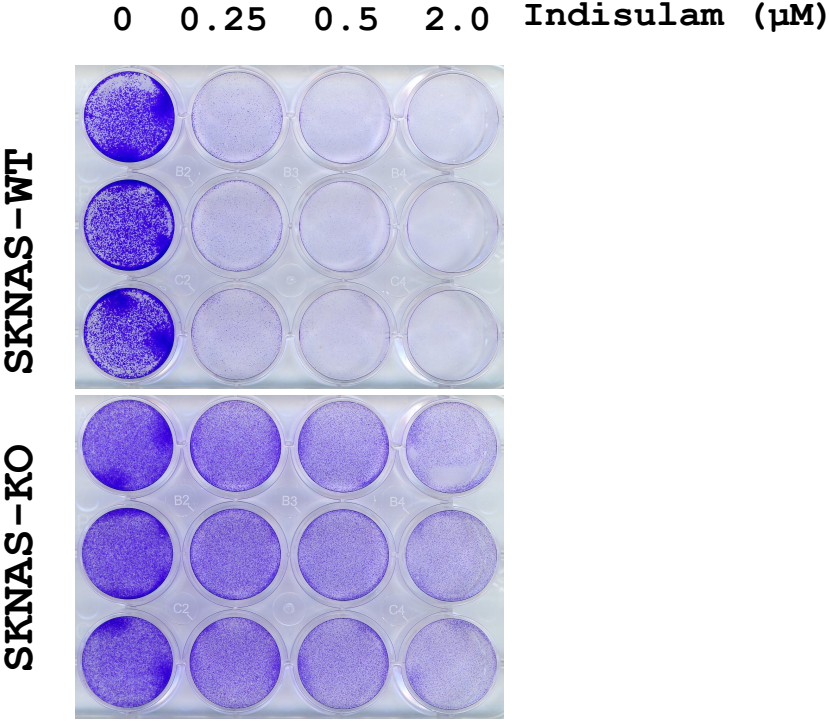

Figure 8l

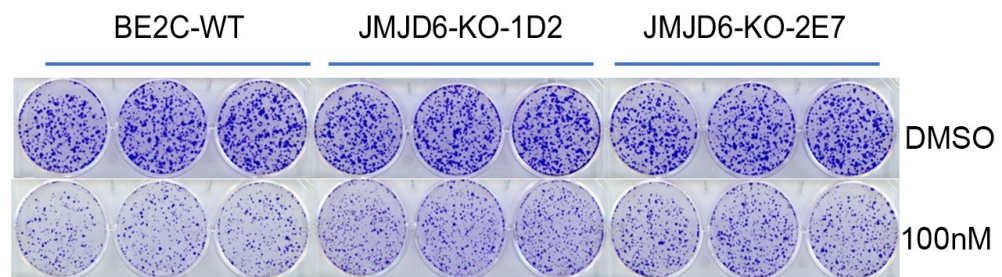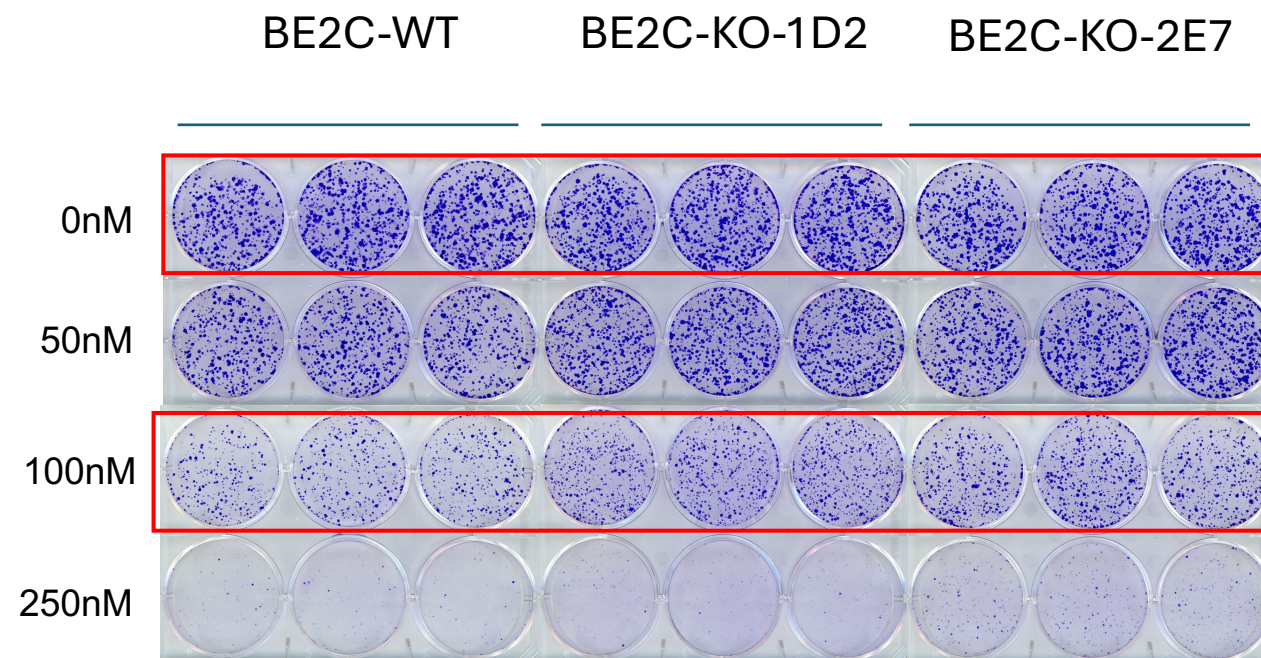

Figure 8K

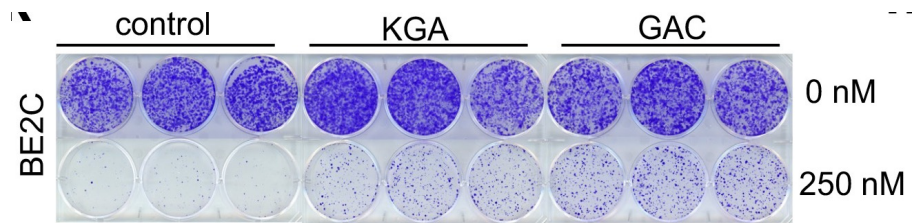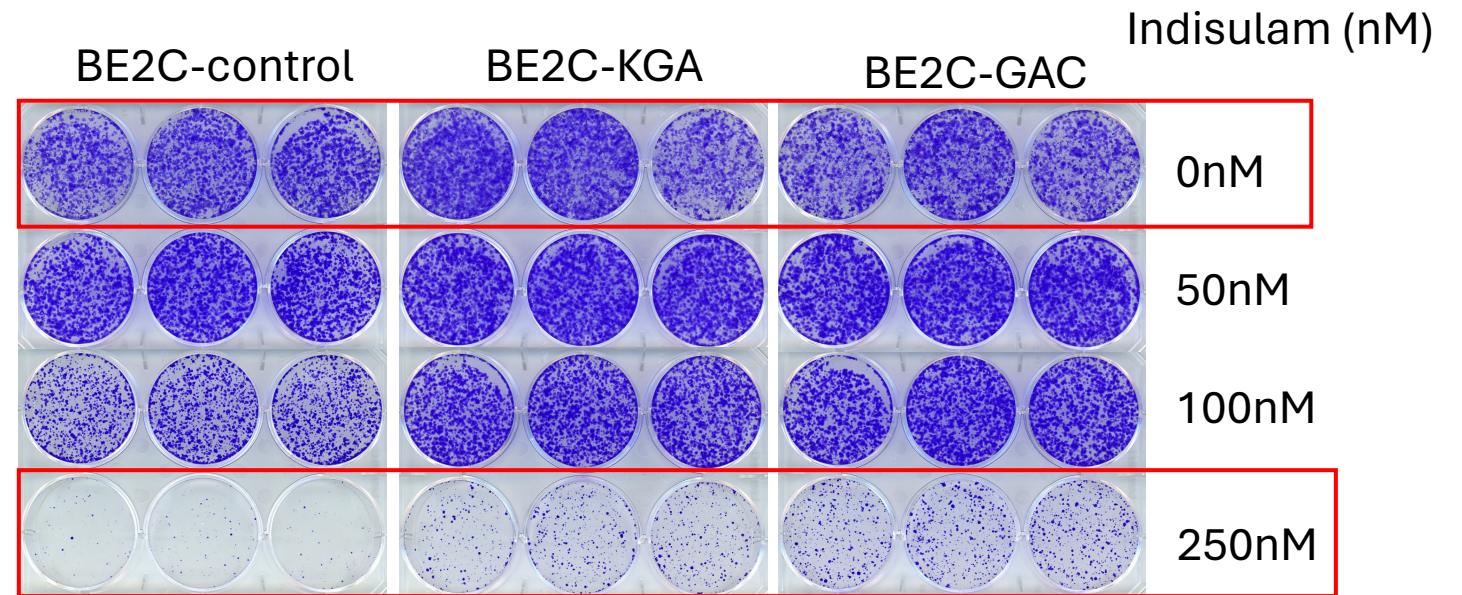

Figure 8i

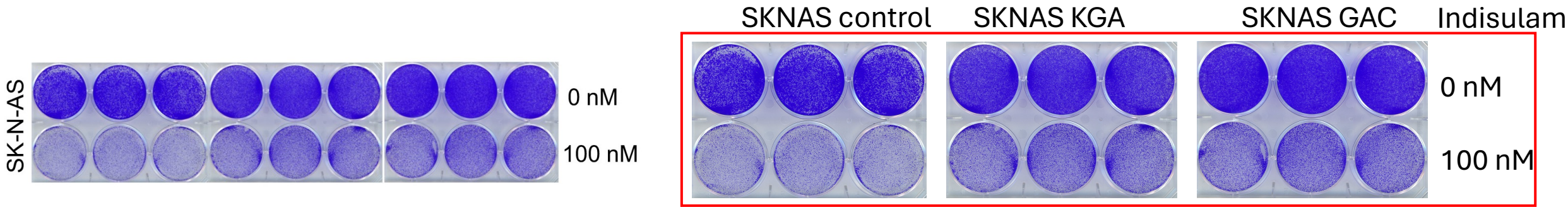

Supplement: Source data 1. [file elife-90993-data1.zip › Figure 8-data source.pdf]
